# EMPATH protocol: Randomized, double-blind trial of an Engaging and Mobile Prism Adaptation Treatment for spatial neglect in Home and rehabilitation settings

**DOI:** 10.1371/journal.pone.0356055

**Published:** 2026-08-17

**Authors:** Catrina MacPhee, Gail Eskes, Sean P. Mackinnon, Anne Sophie Champod

**Affiliations:** 1 Department of Psychology and Neuroscience, Dalhousie University, Halifax, Nova Scotia, Canada; 2 Department of Psychiatry, Dalhousie University, Halifax, Nova Scotia, Canada; 3 Department of Psychology, Acadia University, Wolfville, Nova Scotia, Canada; Neighborhood Physical Therapy, UNITED STATES OF AMERICA

## Abstract

**Background:**

Spatial neglect is a common attentional condition post-stroke linked to poor rehabilitation outcomes. Prism adaptation, though a promising intervention, is not yet widely used clinically due to various barriers to its implementation, including limited accessibility, the repetitive nature of the therapy, and the need for specialized equipment. This protocol aims to examine the effectiveness, feasibility, and acceptability of an engaging, mobile, computerized prism adaptation procedure (Peg-the-Mole) for treating spatial neglect in stroke inpatient and outpatient community settings.

**Methods/design:**

The present study uses a longitudinal, double-blind, quasi-randomized, controlled design. Forty-two individuals with right-hemisphere stroke experiencing mild to severe symptoms of neglect will be randomized into two groups: Use of Peg-the-Mole with 15-degree rightward-deviating prism goggles (intervention) or use of Peg-the-Mole with 5-degree rightward-deviating prism goggles (active control). Participants will complete 10 treatment sessions over 2 weeks, engaging with the Peg-the-Mole procedure for 15 minutes daily. Blinded assessments will take place immediately before and after treatment, as well as at one-month follow-up. The primary outcome measure will be the Star Cancellation conventional subtest of the Behavioural Inattention Test. Secondary outcomes will include additional conventional and behavioural subtests of the Behavioural Inattention Test, one subtest of the Eschenbeck Standardized Activities of Daily Living battery, the caregiver- and self-reported forms of the Halifax Neglect Impact Scale, data measuring feasibility and acceptability, prism adaptation after-effects, the Hospital Anxiety and Depression Scale, and the Fatigue Severity Scale.

**Discussion:**

The Peg-the-Mole procedure has the potential to improve patient recovery and quality of life after stroke due to its gamified, portable features that promote treatment accessibility and adherence. Results from this study will help determine if the treatment can be implemented successfully in inpatient and community settings to improve neglect symptoms and daily living.

**Trial registration:**

Clinicaltrials.gov, Identifier: NCT05595668; registered October 27, 2022.

## Introduction

Spatial neglect is a common and debilitating attentional disorder characterized by an inability to detect, orient to, or respond to stimuli presented on the contralesional side of space [1]. This condition is not due to primary sensory or motor impairments [2,3]. It affects approximately 30-50% of stroke patients, often leading to impaired daily functioning, loss of independence, and slower recovery [4–6]. Symptoms of neglect can lead to serious functional impairment and interfere with mobility, navigation ability, reading, and self-care activities such as eating, dressing, and grooming [4]. It is estimated that approximately 40% of individuals with neglect may go on to develop chronic symptoms [7,8]. Given the debilitating impact of spatial neglect on functional independence, individuals often require increased assistance with activities of daily living (ADLs) which in turn may lead to greater caregiver reliance and burnout [6,9]. Loss of independence in everyday life can also reduce quality of life and may contribute to depression, anxiety, and fatigue [10,11]. Moreover, spatial neglect is associated with longer hospital stays and higher re-admission rates, placing additional strain on healthcare systems [12–14]. Taken together, these challenges highlight the need for easily accessible, effective, and standardized rehabilitation interventions that can enhance functional independence, reduce caregiver burden, and alleviate pressure on the healthcare system.

Various approaches are available for the treatment of spatial neglect, but there is currently no universally accepted standard of care [15–17]. Many existing interventions are resource-intensive, requiring clinician supervision and specialized equipment, and they often lack standardized guidelines [17–19]. Further complicating treatment, up to 64% of individuals with neglect experience a comorbid syndrome called anosognosia, a lack of awareness of their deficits [20]. The presence of both spatial neglect and anosognosia can significantly reduce treatment adherence, often leading to poorer rehabilitation and clinical outcomes [20,21]. Treatment methods are generally divided into two categories, bottom-up (i.e., stimulus-driven) and top-down (i.e., goal-driven) approaches. Given the high prevalence of anosognosia in individuals with spatial neglect, a bottom-up approach is often more appropriate, as these individuals may lack the motivation to develop strategies to address a deficit they do not recognize [20]. Prism adaptation (PA) is a promising bottom-up treatment for spatial neglect. This technique involves wearing prism goggles that shift the visual field rightward, with participants repeatedly pointing towards visual targets. Initially, the visual shift causes rightward pointing errors (i.e., direct effects), but with continued reaching, participants adapt until they can accurately point to the targets again. After 10–15 minutes of prism exposure and reaching, removing the prism goggles results in therapeutic effects called after-effects (i.e., a leftward shift in pointing movements), which may reduce the severity of neglect symptoms and improve daily functioning [22–25]. Thus, PA may have high feasibility as a treatment approach, as it requires little training and does not rely on patient strategy or knowledge of their symptoms [18,26].

Two processes are proposed to underlie the development of after-effects induced by PA as described by Redding and Wallace: strategic recalibration and spatial realignment [27–30]. Strategic recalibration is hypothesized to be an immediate response to pointing errors during PA due to the visual shift, with conscious corrections made to reduce errors in subsequent trials. Conversely, spatial realignment is conceptualized primarily as a slow occurring, unconscious process consisting of the remapping of proprioceptive and visual spatial maps to minimize the discrepancy between the position of the hand and the visual location of the target, leading to the development of after-effects. Strategic recalibration may interfere to some extent with the development of after-effects, although it is thought to still contribute to adaptation during PA [27]. Thus, strategic recalibration during PA is typically minimized using equipment such as an occlusion board to block the patient’s view of their pointing movements to prevent immediate self-correction.

The strength of the prismatic shift is an important factor to consider when evaluating the effectiveness of PA treatment. Randomized controlled trials using sham (i.e., clear) goggles as controls, as well as prisms with smaller shifts (5–6 degrees), found no significant therapeutic effects on conventional and functional outcomes [31,32]. In contrast, studies using prisms inducing a shift of at least 10 degrees have shown promising results, with improvements in both symptom severity and functional outcomes [22,25]. Therefore, a shift of at least 10 degrees is generally recommended to achieve therapeutic effects [17,32].

PA has several advantages as a neglect treatment. It is a bottom-up method requiring minimal strategic processing, making it suitable for individuals with anosognosia, as it does not rely on their awareness of symptoms. PA also has a short administration time, typically requiring 10–15 minutes per day for about ten days [33]. Previous research has demonstrated that PA is effective in reducing symptoms of neglect, with some improvements extending to ADLs [22–24,34]. However, PA has certain limitations that need to be addressed. For example, the repetitive nature of typical PA protocols can reduce patient engagement, and the use of specialized equipment such as an occlusion board, used to block the participant’s view of their hand, is required [35,36]. Clinical supervision in a hospital or research setting is required to ensure the correct procedure is followed, resulting in frequent and repeated travel for each treatment session [36]. Previous research also has found inconsistent evidence for the efficacy of PA, likely reflecting the absence of a standardized protocol and uncertainty surrounding which patients are most responsive to the intervention [17]. Chen et al. (2025) highlight the substantial methodological heterogeneity across PA studies, including variation in prism strength, treatment intensity, arm visibility during adaptation, outcome measurement, and patient characteristics (e.g., time post-stroke). Furthermore, not all individuals with neglect benefit from PA, and factors such as neglect subtypes (e.g., perceptual vs. premotor neglect) and time since stroke onset may impact its effectiveness [26,37]. Thus, clinical recommendations of PA remain tentative due to methodological limitations of previous research and inconsistent outcomes [17,38]. Therefore, although PA may be a feasible treatment for spatial neglect at present, its implementation has been limited to date. The current gaps interfering with the clinical implementation of PA underscore the need for standardized, evidence-based protocols that increase accessibility and engagement, thereby improving long-term functional outcomes in individuals with spatial neglect.

To address these barriers to the clinical application of PA procedures, a home-friendly, game-like PA software called Peg-the-Mole (PTM) was developed. In this protocol, participants wear prism goggles and are prompted to touch as quickly as possible a target bull’s eye held by a cartoon mole presented on an iPad. In comparison to typical PA procedures, PTM requires minimal supervision because the software provides automated instructions, feedback, and data recording, allowing participants to complete sessions independently once trained. The program’s game-like interface enhances engagement and motivation. Importantly, PTM incorporates an adaptive algorithm that adjusts target presentation time based on individual performance, encouraging rapid, ballistic movements and minimizing conscious self-correction during pointing. This algorithm promotes the spatial realignment processes underlying PA while reducing the need for specialized equipment (e.g., occlusion boards). Taken together, these features increase accessibility by enabling standardized, engaging delivery of PA in various settings [36].

Initial testing of PTM was conducted in a sample of healthy young adults comparing it to a typical PA procedure using 15-degree rightward-deviating goggles [36]. Results indicated that prism exposure during PTM yielded adaptation and significant after-effects of equivalent magnitude to those observed with a typical PA procedure. Importantly, participants found PTM to be more enjoyable and engaging than the typical PA procedure. Further PTM studies with both healthy young and older adult samples demonstrated that 15-degree prism goggles induced significantly larger after-effects than 5-degree goggles [39]. Additionally, after-effects induced by PTM have generalized to aspects of wheelchair maneuvering (e.g., a significant reduction in the number of right-sided hits), providing evidence that PTM has the potential to generalize to more functional tasks [40]. More recently, a pilot study examined the feasibility of implementing a desktop version of PTM with stroke patients in an inpatient rehabilitation unit. PTM was administered daily for 10 sessions over two weeks by occupational therapists and the protocol was generally found to be feasible and engaging [41]. In the present study, we adapted PTM for iPad administration to enhance flexibility and accessibility for at-home use.

To support the future clinical implementation of PTM, the primary objective of the present study is to examine PTM’s effectiveness in reducing neglect symptoms as measured by the Star Cancellation task of the Behavioural Inattention Test (BIT) [42]. Short-and longer-term effectiveness will be examined in terms of symptom reduction (i.e., does the PTM PA protocol improve neglect symptoms over time?). We hypothesize that participants who complete 10 sessions of PTM PA treatment using 15-degree rightward-deviating goggles will show a greater reduction in the severity of neglect symptoms immediately after training and at one-month follow up, compared to participants using the PTM protocol with 5-degree deviating goggles.

Secondary objectives include (1) examining treatment effects across additional neglect measures, as well as measures of ADLs, (2) evaluating feasibility and acceptability, (3) examining the magnitude of PA after-effects and the relationship between these effects and neglect severity, and (4) exploring changes in psychological outcomes. First, we will examine whether the 15-degree PTM condition leads to broader improvements in neglect symptom severity across additional neglect assessments, as well as improvements in ADL functioning over time. Second, we will evaluate the feasibility and acceptability of using the iPad-based PA treatment in both inpatient and community settings. Feasibility will be assessed based on compliance with the treatment protocol and schedule, while acceptability will be evaluated through patient feedback. PA after-effects (mean difference in degrees of visual angle from pre- to post-PTM) will be measured after each treatment session. We will examine whether the 15-degree condition produces larger after-effects compared to the 5-degree condition. Additionally, we will investigate whether the magnitude of PA after-effects predicts neglect severity. The relationships between neglect severity and psychological variables, including anxiety, depression, and fatigue, as well as changes in these psychological outcomes following participation in the PTM protocol will also be explored. Although fatigue, anxiety, and depression are prevalent post-stroke [43,44], their association with spatial neglect remains underexplored, and these secondary analyses will provide insight into the broader impact of PTM and whether improvements in neglect symptoms may be associated with changes in psychological outcomes and fatigue severity.

## Materials and methods

### Study design and setting

The current study uses a longitudinal, quasi-randomized, double-blind, controlled design to compare the effects of using the PTM protocol with 15-degree versus 5-degree prism goggles, each provided in addition to usual care. The study is registered at Clinicaltrials.gov (NCT05595668) and follows the Standard Protocol Items: Recommendations for Interventional Trials (SPIRIT) guidelines [45]. The present study has received ethics approval from the Nova Scotia Health Research Ethics Board (REB File #:1028679; dated March 6^th^, 2023). All participants will provide written informed consent prior to participation.

**Fig 1:**
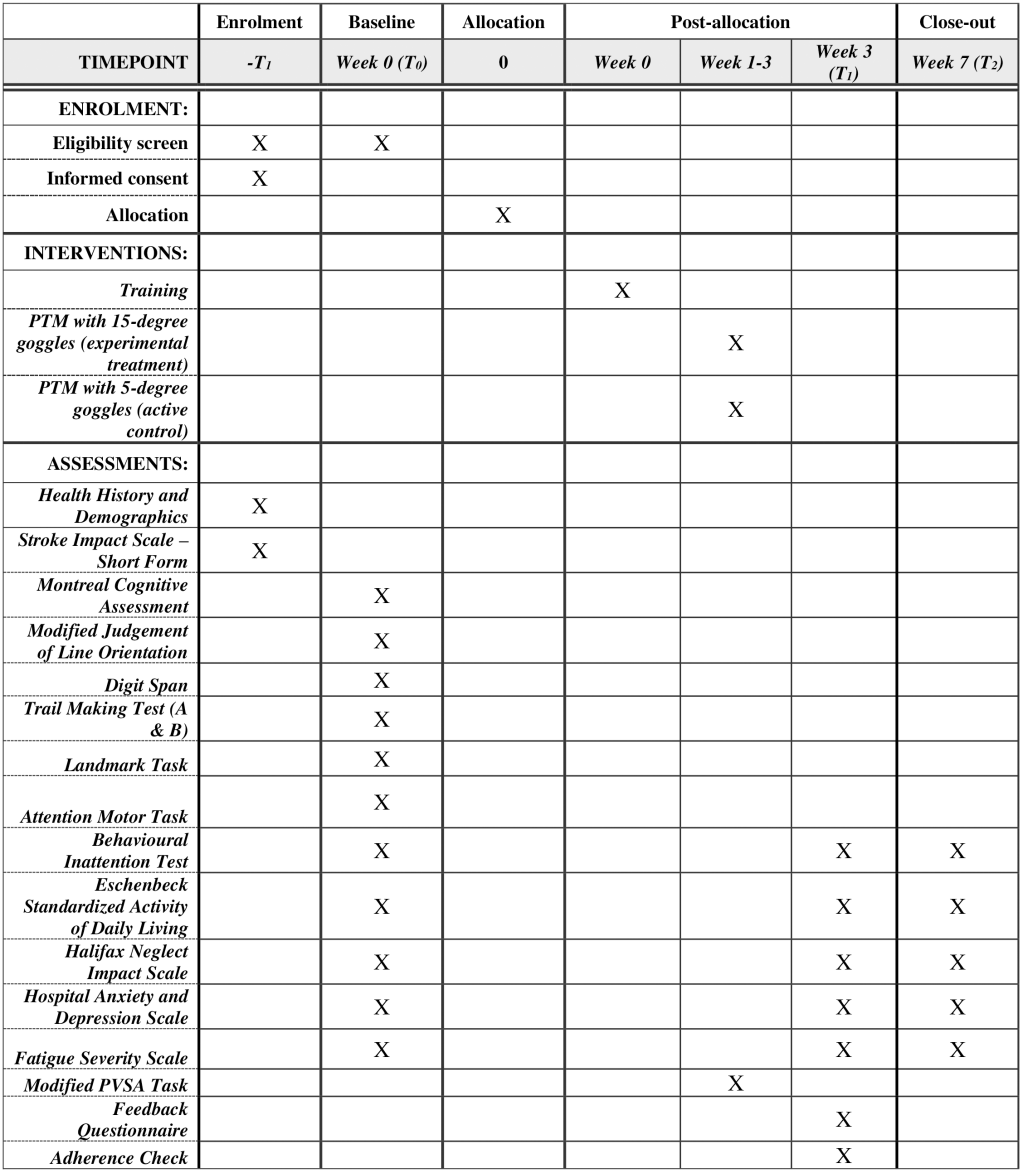
EMPATH study schedule (SPIRIT guidelines).

**Fig 2:**
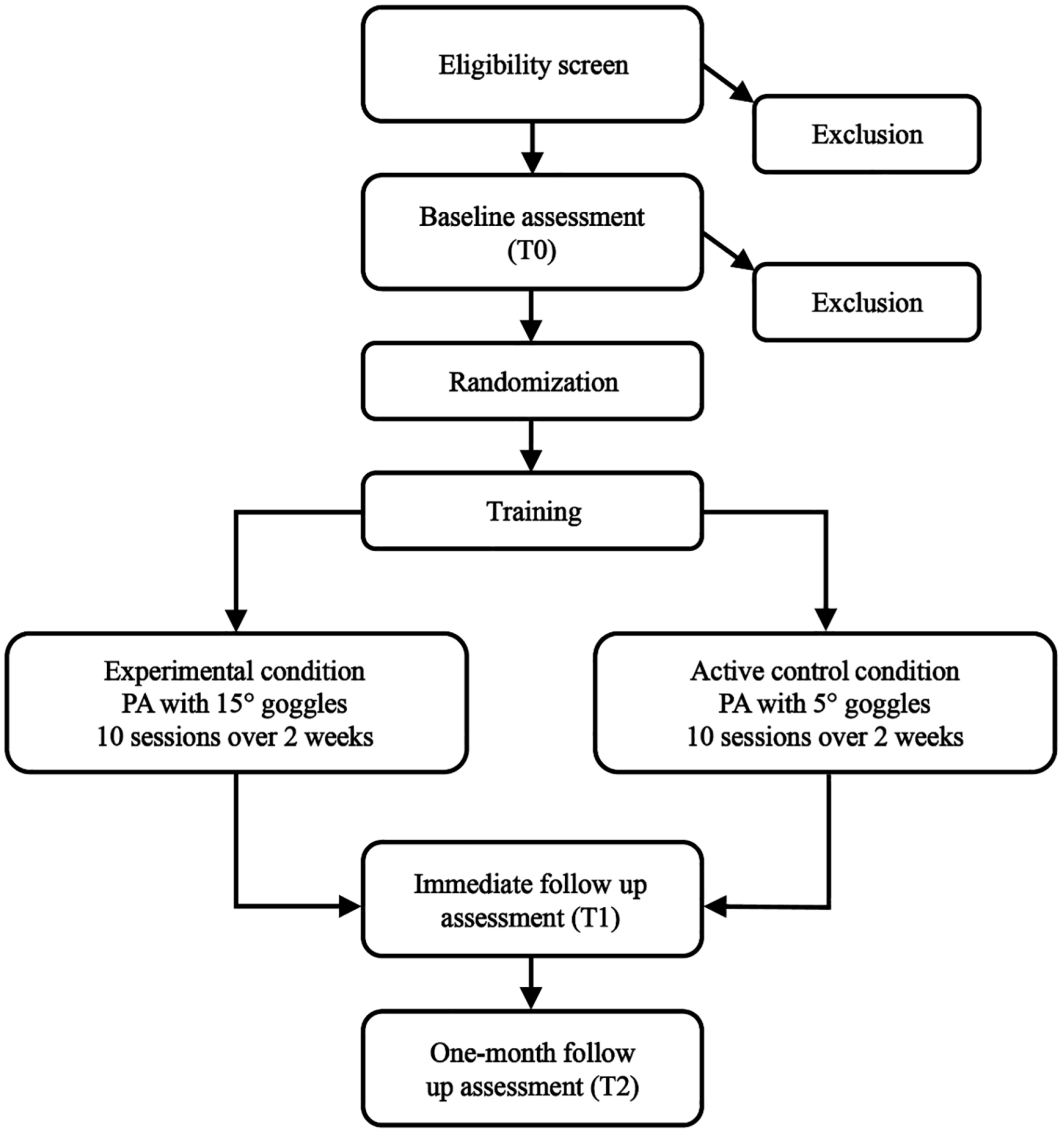
Study timeline. After the baseline assessment (T0), eligible participants are randomized (stratified by neglect severity) and undergo a 10-day treatment over two weeks. Follow-up assessments occur within one week and one month of finishing treatment (T1, T2, respectively).

Participants will be initially screened for inclusion and exclusion criteria before undergoing a baseline assessment (see criteria below). Participants currently experiencing symptoms of spatial neglect, as determined by the baseline assessment, will be randomized, stratified by neglect severity, to one of two groups: experimental condition (PTM with 15-degree rightward-deviating goggles) or active control condition (PTM with 5-degree rightward-deviating goggles). Each participant will be assigned an “Assessor”, responsible for all assessments, and a “Trainer”, responsible for overseeing the PTM protocol training and scheduling. Trainers will observe participants during the first PTM session to ensure proper adherence to the protocol. Participants will then complete 10 daily training sessions over two weeks (5 days per week), with each session lasting approximately 15 minutes. Assessments will be conducted at 3 time points: baseline (T0), within one week after the final treatment session (T1; immediate follow-up), and within 4–6 weeks after the final treatment session (T2; one-month follow-up). After each PTM training session, after-effects will be measured, and the treatment and after-effects data will be stored on the iPad for later download. At T1, participants will independently set up and complete training in front of their Assessor to confirm their ability to follow the protocol as trained. The study will be conducted both in the community and across various hospital sites in Nova Scotia, Canada. Data collection is currently ongoing. This trial is open to recruitment from June 20^th^, 2024, to August 31^st^, 2027. Data collection is anticipated to be complete by September 2027, and results are anticipated by August 2028. See Fig 1 and Fig 2 for the study schedule and timeline, respectively.

### Study population

Participants with right-hemisphere stroke will be recruited from several sites in Nova Scotia: the Acquired Brain Injury Program at the Nova Scotia Rehabilitation and Arthritis Centre, the Early Supported Stroke Discharge Team, and the Acute Stroke Program of the Valley Regional Hospital. Recruitment is also open to the community. Participants may be referred to the study by a clinician or directly recruited from the community by the research team through advertisement and stroke support group visits. Study staff will visit participants for training and assessments, either at their home or in inpatient units, depending on each participant’s current admission status. For participants who enroll while in an inpatient unit but are discharged before completing the study, a hybrid approach will be implemented, allowing them to complete the remaining aspects of the study at home.

#### Inclusion criteria.

To be eligible for the baseline assessment, participants must: 1) have experienced a right-hemisphere stroke; 2) be willing and able to hear about the study and consent to participate; 3) be medically stable; 4) have self-reported normal to corrected-to-normal visual acuity; 5) be able to point with the unaffected limb; and 6) reside within a two-hour radius of Halifax, which ensures accessibility of resources and proximity to the research team. The baseline assessment will be used to confirm symptoms of neglect before randomization into the treatment phase. Neglect will be defined by impaired performance on at least 2 subtests of the BIT [42] and/or at least two ratings of “often” on the Caregiver version of the Halifax Neglect Impact Scale, indicating frequent impacts of neglect symptoms on ADLs.

#### Exclusion criteria.

Participants will be excluded if they have any other existing neurological disorder (e.g., dementia, multiple sclerosis, epilepsy).

### Sample size

The sample size for this study was determined using the Superpower [46] package in R 4.5.3 software. The primary outcome measure for this study is the total score on the Star Cancellation conventional subtest of the BIT, and the primary analysis strategy will be analysis of covariance (ANCOVA). We used the threshold for a clinically significant effect size measure proposed by Székely et al. (d = 0.61) [47]. A test-retest correlation during treatment of *r* = 0.63 (R^2^ = 0.40), was estimated using raw data from the supplementary materials of Vilimovsky et al. [48], using their results from the Bells cancellation test as a reasonably close proxy. Thus, assuming an alpha of 0.05, 80% power, d = 0.63, and a R^2^ for covariates of.40 requires a sample size of 27 per group. Because of local feasibility concerns when studying this population, we believe it is unlikely it would be possible to collect more than 25 participants per group. Thus, we lowered our threshold for statistical power to 70%, which requires a minimum sample size of 21 per group as our target. Although 80% power is commonly treated as a benchmark, power between 70% and 90% remains within accepted norms [49]. Consistent with recommendations that sample size in constrained clinical populations should reflect the maximum feasible recruitment under real-world conditions [50], our target sample also takes into consideration practical feasibility. The R script with our power analysis is included in the supporting information materials S3 File.

### Randomization

Covariate-adaptive randomization, as described by Sella et al. [51], will be used to assign participants to treatment conditions. Randomization is stratified based on neglect severity, assessed using the BIT [42]. Specifically, participants will be considered as having “mild” neglect if they fail fewer than 3 subtests on the BIT, or when only functional symptoms are reported. Participants will be considered as having “moderate to severe” neglect when they fail 3 or more subtests on the BIT. Severity classification will be completed by a blinded assessor. Participants will be assigned to one of two groups in a manner that balances neglect severity between groups, to control for potential baseline differences in symptom severity. Randomization will be conducted by a member of the research team who is not involved in data collection or analysis and provided to the assigned Trainers immediately before the “training” visit (i.e., home or inpatient visit #2).

### Blinding

The Trainer assigned to each participant will not be blinded to the treatment condition, as they are responsible for training participants on the protocol and they will handle the goggles (i.e., 5- or 15-degree). To minimize trainer influence, all trainers receive standardized training from the same supervisor and must demonstrate competence before interacting with participants. Trainers follow detailed, manualized protocols to ensure procedural consistency across both trainers and sessions, and training tasks are fully computerized with automated instructions and feedback. These steps substantially reduce the potential for bias. Assessors will remain blinded to the treatment condition, and participants will not be informed of which treatment they are receiving. As previous research has shown that 15-degree goggles induce significantly larger after-effects than 5-degree goggles when using PTM, 5-degree goggles will be used as an “active control” [39]. Using 5-degree deviating goggles as an “active control” rather than the typical sham (i.e., clear) comparator preserves the double-blind design of the present study. Upon completion of the study, both participants and their Assessors will be asked to indicate which treatment condition they believe was assigned.

### Intervention

#### Equipment.

For PTM training, participants will receive Welder goggles fitted with Fresnel prisms inducing a rightwards optical shift of either 5- or 15-degrees (Insight Optometry Group, Halifax, Canada). The PTM software will be administered using a case-protected 9^th^ generation Apple iPad (64GB;10.2-inch LED-backlit multi-touch display with IPS technology; resolution: 2160 x 1620 pixels; iPad width: 21 cm). A wireless QWERTY computer keyboard (OMOTON Ultra-Slim Wireless Bluetooth) will be used with the iPad.

#### Experimental condition.

PA will be administered using the PTM software, which displays pointing targets, records touch locations and provides feedback on accuracy and response time (in milliseconds). Participants are seated in front of an iPad, centered with the screen, at a distance allowing them to touch the screen with a slightly bent arm. While wearing 15-degree rightward-deviating prism goggles, participants are instructed to touch a target bull’s eye held by a cartoon mole on an iPad, as quickly as possible. To initiate each trial, participants hold down the spacebar on the QWERTY keyboard with their dominant hand. When the mole appears in one of 9 locations on a 3x3 grid, participants release the space bar and attempt to touch the target as quickly as possible. After each response, the mole provides visual and auditory feedback on accuracy and response time.

To calculate the initial target presentation time for the exposure task for each participant, 10 practice trials will be administered without the use of prism goggles. Practice trials may be repeated as necessary. The mean response time over last the 10 practice trials will be used as the initial target presentation time for the exposure task. The exposure task, lasting approximately 15 minutes (depending on response speed), involves 117 trials in total (13 trials for each of the 9 target locations). Between tasks (e.g., practice trials and exposure task), written and audio-recorded instructions are provided to guide participants through each step (e.g., “put goggles on”).

The PTM software uses an algorithm that adjusts the target presentation speed based on individual performance, promoting ballistic movements and minimizing self-correction in pointing movements. The software uses an algorithm to ensure performance accuracy is maintained by manipulating target presentation time based on the participants’ performance. To further encourage ballistic pointing movements and enhance engagement in the task, participants receive feedback when they reach a new timing level. The PTM software records participants’ pointing errors in degrees of visual angle (DVA).

Participants will be asked to complete 10 training sessions over a two-week period. To enhance and monitor treatment adherence, participants will be asked to fill out daily training logs, and their assigned Trainer will make weekly check-in phone calls. For additional support, participants will have access to a phone number and email for troubleshooting. The PTM intervention will be administered in addition to usual care.

#### Active control condition.

The active control condition follows the same procedure as the experimental condition, with one key difference: participants in the control group wear 5-degree rightward-deviating goggles while completing the exposure task (i.e., PTM). Participants complete the exposure task (PTM) while wearing these 5-degree goggles, providing a mild visual shift that serves as an “active control” to maintain double-blind conditions without delivering the therapeutic effect expected from the 15-degree shift.

### Data collection and management

All research staff, including Assessors and Trainers, will receive standard training on data collection methods from the same supervisor. Study personnel consist of graduate-level clinical psychology students and supervised undergraduate students. All students must demonstrate competence in all tasks required for the “Trainer” and “Assessor” roles (e.g., administration of assessments) prior to data collection. Study personnel follow manualized protocols throughout the study.

Consent forms, participant demographics and health history, training logs, and data summaries will be securely stored using the Research Electronic Data Capture (REDCap) system hosted by Nova Scotia Health. Each participant will be assigned a unique identification number to ensure data de-identification. Other electronic data files will be stored on password protected computers or saved on a secure lab network behind a firewall. Access to electronic data is restricted to authorized personnel. Paper files will be stored in a locked laboratory with access limited to authorized personnel. If a participant withdraws from the present study or their participation ends early, all data collected up to the date they withdraw their consent and/or their study participation ends, will be maintained in the study records to be included in the study related analyses.

### Measurements

#### Screening data.

Screening data will be collected with a health history questionnaire after obtaining informed consent and before the baseline assessment. The questionnaire includes demographic information (e.g., age, gender, education level) and health history, such as any relevant health and psychiatric conditions. Additionally, the Stroke Impact Scale Short Form (SF-SIS) [52] will be administered to assess stroke impact across various dimensions such as mobility and ADLs.

#### Baseline data.

After initial screening, a baseline assessment (T0) will be conducted. The purpose of the baseline assessment is to describe the cognitive profile of the sample. This assessment includes: confrontation visual field testing, Montreal Cognitive Assessment (MoCA) [53], a shortened version of Judgement of Line Orientation (JLO) [54,55], Digit Span (forward and backward) [56], Trail Making Test (A and B) [57], abbreviated Landmark Task [58], and the Attention Motor Task (i.e., a reaching task designed to characterize neglect subtype) [59].

#### Primary outcome.

The Star Cancellation conventional subtest of the Behavioural Inattention Test (BIT) [42] will serve as the primary outcome and will be measured at three time points: T0 (baseline), T1(immediately post-intervention), and T2 (one-month post-intervention). This measure assesses changes in performance from pre- to post-prism training and was selected as the primary outcome due to its greater sensitivity in comparison to other paper-and-pencil tasks [42,60]. The Star Cancellation subtest requires patients to cancel small stars among distractors on a page that is placed in their center of body. The scores range from 0–54, with scores at or above 52 as the “acceptable range”. The subtest has excellent test-retest reliability [61]. Additionally, compared to other measures of neglect, previous research has found that the Star Cancellation subtest is the best predictor of functional outcome as assessed by an ADL scale [62].

#### Secondary outcomes.

Secondary outcomes will include the remaining conventional subtests of the BIT [42], three behavioural BIT subtests (menu reading, article reading and picture scanning) [42], one subtest of the Eschenbeck Standardized Activity of Daily Living Test (SADL; *filling out a form*) [63], the Halifax Neglect Impact Scale (self- and caregiver-reported measures assessing the frequency and impact of neglect symptoms on ADLs), the Hospital Anxiety and Depression Scale [64] and the Fatigue Severity Scale [65]. Each of these secondary outcomes will be administered at all three assessment time points.

Feasibility and acceptability data will also be collected at T1 (i.e., within one week of training) via a Patient Exit Interview, using a modified version of the Intrinsic Motivation Inventory [66] which includes 38 Likert scale questions and 8 open-ended questions about usability and enjoyment of the PTM software.

To measure PA after-effects, a modified Proprioceptive-and-Visual Straight-Ahead (mPVSA) task (described below) will be administered immediately after each training session.

#### Measurement of after-effects.

An mPVSA outcome task will be used to assess after-effects before and after each treatment session, without the use of prism goggles. The mPVSA task was developed specifically to accommodate the home-based nature of the protocol, eliminating the need for an occlusion board, which is often unavailable in home and rehabilitation settings. During the mPVSA task, participants will view a cartoon bull’s eye at one of two untrained locations on the central horizontal row of the 3x3 grid displayed on an iPad screen. This setup allows for the examination of the generalization of after-effects to untrained spatial locations. Participants will be asked to memorize the target location and then press the spacebar on a QWERTY keyboard with the index finger of their dominant hand. When the spacebar is pressed, the target disappears behind a pixelated colour screen. Once the colour screen disappears, participants will use the same index finger to touch the screen where they last saw the target. The pixelated colour screen serves as a visual mask, supporting memory-guided pointing without the need for an occlusion device. Auditory cues will confirm the participant’s touch response for each trial. Each session will include ten trials administered immediately before and after the PTM exposure task, with relevant instructions provided both in writing and as audio recordings. Mean after-effects correspond to the difference in endpoint accuracy (in DVA) from pre- to post-PTM. Touch location on the iPad screen is recorded in real time. As the task is self-administered, potential sources of measurement error include premature responses and mis-registered screen touches which are mitigated through auditory/visual instructions and feedback. Additionally, posture and viewing position differences between participants are minimized through a standardized set-up completed by the Trainer during the initial session. The Trainer tapes the iPad position on the table and provides a pre-cut string so the participant can measure the appropriate viewing distance from the iPad before each training session. Protocol adherence is evaluated at the end of the two-week training period.

### Statistical analyses

Given the heterogenous nature of neglect, deficits may not be captured by a single task; therefore, a battery of subtests was selected to assess functioning across multiple areas (e.g., functional and paper-pencil tasks). Subtest-level analyses will capture deficits and improvements across different areas of functioning. Note that the study has a single primary outcome (Star Cancellation subtest of the BIT); thus, the strongest evidence of the trial’s efficacy will be determined from this a-priori outcome. Secondary outcomes will have weaker evidential value but may remain useful for future meta-analyses and to inform future research.

To assess the effectiveness of the PTM intervention on neglect severity over time, data will be analyzed using analysis of covariance (ANCOVA) using generalized linear modelling with the glm() function in R software. We are interested primarily in two mean differences: (a) changes from pre-PTM (T0) to immediate post-PTM (T1) and (b) changes from pre-PTM (T0) to 1-month post-PTM (T2). Because mean differences from T1 to T2 are assuredly smaller than comparisons to baseline, our study would be underpowered to detect them, so we have elected to calculate only the primary comparisons of interest. In the first ANCOVA analysis, the outcome will be at T1, and predictors will include T0 scores and time post-stroke as covariates, and Condition (5-degree vs. 15-degree goggles) as a between-subjects factor. In the second ANCOVA analysis, the outcome will be at T2, T0 scores, time post-stroke, and follow-up assessment timing will be used as covariates, and Condition will again be a between-subjects predictor. In the second analysis, follow-up assessment timing will be included to assess whether differences in timing for post-treatment assessments impacts treatment outcomes.

The primary outcome (Star Cancellation conventional subtest of the BIT) produces integer data and likely has a left skewed distribution, with an inflation of perfect scores. Thus, analyzing the data with methods suitable for count data may provide a better fit to the data. Since most commonly available methods are intended for use with right-skewed data, the scores will be reflected (i.e., re-scaled by subtracting 54 then taking the absolute value of scores) such that the scale will indicate the number of misses instead of the number of hits, producing right-skewed data. Because the population distribution of scores is unknown, our approach will be to analyze the data two ways (a) with a gaussian distribution and an identity link, which is equivalent to a standard ANCOVA and (b) assuming a negative binomial distribution with a log link, which assumes a right skewed distribution with overdispersion (i.e., mean > variance). We will compare the Bayesian Information Criteria (BIC) between the two models, and if the ΔBIC is > 2.0 we will prefer the model with the smaller BIC [67]. If the change in ΔBIC is inconclusive, we will prefer the gaussian (i.e., normal) model because it is more parsimonious. Analyses on primary and secondary outcomes will generally follow the same procedures, excepting that negative binomial models will not be run on outcomes unsuitable for such approaches (e.g., non-integer data). We will report unstandardized adjusted mean differences and the change in ΔR^2^ values when adding condition as a predictor in a second step after covariates as effect sizes. Technically-minded readers can refer to the R script posted in our supporting information materials S3 File for further details of how analyses will be conducted.

To examine feasibility and acceptability, descriptive statistics and mean ratings from participant exit interviews will be examined. Additionally, the number of completed intervention sessions will be tallied for each participant, and the mean proportion of treatment session completion will be calculated. The protocol will be deemed feasible if at least 80% of treatment sessions are successfully completed across participants.

To examine the impact of after-effects, a single average score over the two weeks of prism adaptation will be calculated. Then, we will examine the relationship between these mean after-effects scores and condition using an unequal-variances t-test. We will also explore the Pearson correlation between mean after-effects scores and the primary outcome.

An intention to treat analysis will be completed. We will also examine outcomes of participants who complete at least 80% of training and assessment components. Given the nature of our follow-up procedures, we expect low dropout. If missing data < 5%, listwise deletion will be used (complete cases) as when there is minimal missing data, more advanced strategies generally perform equivalently to listwise deletion [68]. If there is substantial missing data, it will be handled using multiple imputation using the Jomo [69] and mitml [70] packages in R software. In such a case, we will evaluate whether missing data is associated with any measured variables to incorporate into the missing data model.

All protocol deviations will be documented and reported to the principal investigator. Deviations that could affect participant safety will be promptly communicated to the ethics board. All deviations will be reviewed to assess impact, and corrective and preventative actions will be implemented as necessary to minimize recurrence.

### Safety considerations

The training intervention is non-invasive, and no serious adverse events are anticipated. However, participants may experience frustration or mental fatigue. To ensure participant well-being, they will be closely monitored throughout the study, and any adverse events will be documented and promptly addressed. Participants will continue to receive their standard stroke rehabilitation care alongside their involvement in this study. In the unlikely event of a significant decline in a participant’s condition or upon their request to withdraw, they will be immediately withdrawn from the study, and data collection will cease. If a participant becomes ill or injured as a direct result of participating in this study, necessary medical treatment will be available to the participant at no additional cost.

### Knowledge translation and dissemination

Our team will actively engage in a range of knowledge translation activities to disseminate our findings. These will include presenting results through media outlets, conducting talks for stroke support groups, participating in relevant conferences, and clinical rounds at partnered hospitals. The study protocol will be available for public access. However, given the clinical nature of the dataset collected in the present study, only group-level data will be available upon request. Protocol modifications that have ethical considerations will be reported to the Nova Scotia Health Research Ethics Board and study participants. All protocol modifications will be reported during the final publication of study results.

## Discussion

Spatial neglect is a common disorder post-stroke linked to poor rehabilitation outcomes and reduced independence in ADLs. There is currently an unmet need for an accessible, effective, and feasible intervention for the treatment of this condition. The present ongoing study aims to address this gap by conducting a randomized, controlled, double-blind, clinical trial to evaluate the effectiveness of using PTM with 15-degree deviating prism goggles, compared to an active control condition. The study will assess both short-and longer-term treatment effects on neglect severity.

This clinical trial uses an innovative protocol that allows for treatment in both inpatient and home settings, which enhances accessibility for individuals with spatial neglect. The gamified aspects of PTM are designed to increase engagement and adherence, potentially improving participants’ recovery and quality of life. Results from this study will provide valuable insights into the feasibility and acceptability of implementing PTM in diverse settings, as well as identifying patient characteristics associated with a positive response to treatment. Ultimately, if the intervention is feasible and effective, it could contribute to improved continuing care, enhanced quality of life after stroke, and increased access to rehabilitative services for those with neglect.

There are several limitations of the present study design and implementation that are important to note. First, although conducting treatment and assessments in participants’ homes and inpatient units enhances accessibility and adherence, data collection within specific geographic regions may limit the generalizability to the broader population of stroke survivors given the heterogeneity of spatial neglect. Second, although all participants receive standardized training on PTM use, differences in familiarity, confidence with, and attitudes toward technology may influence treatment adherence, particularly among older adults [71]. Third, despite procedures implemented to ensure standardization (e.g., standardized set-up, manualized training, written instruction sheets), the home-based context introduces potential variability between sessions and participants due to environmental factors (e.g., distractions) and user error. Finally, the relatively short follow-up period limits the ability to draw conclusions about the durability and long-term maintenance of treatment effects. These limitations will inform refinements to future iterations of the protocol to optimize standardization and scalability.

From a clinical perspective, previous work has emphasized the broader therapeutic value of structured physical therapy and exercise in improving neuropsychological outcomes. Building on this, our study highlights how game-like, home-friendly approaches can enhance engagement and rehabilitation effectiveness in disorders such as spatial neglect [72]. If PTM proves to be an effective, feasible, and engaging treatment for spatial neglect, findings from the present study could help inform clinical practice by promoting scalable, accessible, home-based rehabilitation strategies. Additionally, the results may guide the development of evidence-based recommendations for spatial neglect care after stroke. Consistent with recent work on other at-home interventions (e.g., ecological PA, telerehabilitation, and virtual reality interventions) [73,74] the present study highlights the importance of accessible, technology-supported rehabilitation strategies to improve patient engagement in post-stroke rehabilitation and long-term functional outcomes.

Future research should examine the optimal dose of PTM, including session length, frequency, and total number of sessions needed to maximize therapeutic benefits [33]. Long-term maintenance of treatment effects should also be examined. Additional studies should also evaluate the scalability, cost-effectiveness, and integration of PTM within existing stroke rehabilitation pathways, including implementation across multiple clinical and home-based settings.

## Supporting information

S1 File Ethics protocol. (PDF)

S2 File SPIRIT checklist. (PDF)

S3 File R Script. (R)
