# Supplementary material for: EMPATH protocol: Randomized, double-blind trial of an Engaging and Mobile Prism Adaptation Treatment for spatial neglect in Home and rehabilitation settings
Source: PLoS One. 2026 Aug 17;21(8):e0356055. doi: 10.1371/journal.pone.0356055 (PMC13480593; doi:10.1371/journal.pone.0356055)
Supplement: Supplementary file 1 [file pone.0356055.s001.pdf]

RESEARCH PROTOCOL

CLINICAL STUDY REGISTRATION NUMBER: NCT05595668

STUDY TITLE: Feasibility and effectiveness of a new computerized treatment protocol for spatial neglect post stroke during inpatient and outpatient rehabilitation

SHORT NAME: Engaging Mobile Prism Adaptation Treatment at Home (EMPATH)

INVESTIGATORS

Dr. Anne Sophie Champod, Ph.D., R. Psych. (Principal Investigator)
Associate Professor
Dept. of Psychology, Acadia University
Dept. of Psychiatry and Psychology & Neuroscience, Dalhousie University

Dr. Gail Eskes, Ph.D., R. Psych. (Co- Investigator)
Professor
Dept. of Psychology and Neuroscience, Faculty of Science
Faculty of Medicine (Psychiatry and Neurology), Dalhousie University

Catrina MacPhee, M.Sc.
Ph.D. Candidate
Dept. of Psychology and Neuroscience, Dalhousie University

Matthew Miller, M.Sc.
Occupational Therapist
Occupational Therapist, Acquired Brain Injury Services
Nova Scotia Rehabilitation and Arthritis Centre

Dr. Jasmine Aziz, Ph.D.
Dept. of Psychology and Neuroscience, Dalhousie University

Emily Wildeboer, B.Sc. (Hons)
Ph.D. Student
Dept. of Psychology and Neuroscience, Dalhousie University

Momina Raja, B.Sc. (Hons)
Ph.D. Student
Dept. of Psychology and Neuroscience, Dalhousie University

Hayley Green
B.Sc. Student
Dept. of Psychology, Acadia University

Ian Armstrong
B.Sc. Student
Dept. of Psychology, Acadia University

Joelle Safatli
B.Sc. Student
Dept. of Psychology and Neuroscience, Dalhousie University

Audra Gezen
B.Sc. Student
Dept. of Psychology and Neuroscience, Dalhousie University

SOURCE OF SUPPORT

Nova Scotia Health Research Fund

OVERVIEW

Spatial neglect is a common disorder after stroke which leads to problems with noticing or responding to information from the left side of the body or space. This condition has been linked to reduced independence in a wide range of daily life activities including eating, dressing, and mobility. There is currently no standard of care for spatial neglect, but prism adaptation (PA) is a promising treatment. During PA treatment, participants wear goggles fitted with prisms that shift vision, and point repeatedly at targets presented on a screen for about 15 minutes. After PA, when the goggles are removed, participants now pay more attention to the previously neglected left side of the world. However, there are limitations to using PA in standard clinical care, as PA requires daily exposure for at least two weeks, yet access to equipment and supervision is limited. To solve this problem, we developed a new, game-like, iPad administered, PA treatment (Peg-the-Mole, PTM) that can be used at home or in inpatient settings without the need for daily in person therapist set-up and supervision. Acceptability of a computerized version was confirmed in a pilot study of 10 participants in a rehabilitation setting. However, the iPad version of PTM has not yet been tested at home or in inpatient rehabilitation settings to treat neglect. The present study will examine the feasibility and effectiveness of translating our new, iPad administered, PA procedure to the inpatient and outpatient settings. Stroke participants with neglect will use PTM for 15 minutes a day for 2 weeks at home (outpatient) or in a stroke rehabilitation centre (inpatient). For feasibility, we will examine how easy it is to learn, use, and complete the protocol and gather feedback on how engaging it is. For effectiveness, we will measure changes in neglect symptoms and signs and self-reports of benefits to daily life. The results will help us determine if this innovative treatment can be used successfully in inpatient and outpatient settings and who is most likely to benefit. These findings should aid in achieving more effective and accessible continuing care and enhance the well-being of stroke participants by improving rehabilitation outcomes after stroke.

Background and Rationale

Spatial neglect is a common debilitating neurological disorder that typically results from unilateral brain injury, such as a stroke. This condition is characterized by an inability to detect, orient, or respond to stimuli presented on the contralesional side of space (Li & Malhotra, 2015). The deficits associated with neglect are not caused by

impairments in primary sensory or motor functioning (Langer et al., 2019). Approximately 50% of right brain-damaged stroke participants will experience spatial neglect, leaving many with impaired daily functioning, a loss of independence in everyday life, and long recovery periods (Chen et al., 2015; Ogourtsova et al., 2017). Specifically, individuals with spatial neglect will often face difficulties with mobility, reading, and self-care activities such as eating, dressing, and grooming (Basagni et al., 2019; Mark, 2003). Previous research has found that up to 65% of participants with spatial neglect will also experience a comorbid syndrome called anosognosia, which refers to a lack of awareness of deficits (Grattan et al., 2018). The combination of spatial neglect and anosognosia will often lead to a reduction in treatment adherence, and consequently, to poorer rehabilitation outcomes (Appelros et al., 2003). Therefore, spatial neglect often results in poor clinical outcomes as well as a loss of quality of life (Dai et al., 2014; Nys et al., 2006).

Many approaches are available for the treatment of spatial neglect, however, there is no universally accepted standard of care (Riestra & Barrett, 2013). Treatments can be divided into two main categories, bottom-up (i.e., stimulus-driven) and top-down (i.e., goal-driven) approaches. Given the significant number of neglect participants who also experience anosognosia, a bottom-up approach is a more appropriate choice for treatment as these individuals are often unmotivated to develop strategies to improve a deficit that they do not recognize (Appelros et al., 2003). Prism adaptation (PA) is a promising bottom-up treatment for spatial neglect. PA involves wearing prism goggles that shift the visual field rightward. While wearing these goggles, the participant points repeatedly towards visual targets. Rightward pointing errors (i.e., direct effects) are initially observed during prism exposure. With continued reaching, participants will then quickly adapt to the visual displacement until they can accurately point to the targets. Once the prism goggles are removed after (approximately 15 minutes of exposure), adaptation will have resulted in a leftward shift in pointing movements from pre- to post-exposure (i.e., after-effects). After-effects are due to a leftward shift in attention and more movements in the left hemispace, which can lead to improvements in daily functioning and a reduction in the severity of symptoms of neglect (Mizuno et al., 2011; Rusconi & Carelli, 2012; Serino et al., 2009; Spaccavento et al., 2016).

PA is a promising neglect treatment for several reasons. First, PA requires less strategic processing as it is a bottom-up treatment, and therefore, does not depend on the participants' awareness of their own symptoms, making it ideal for those with anosognosia. PA also has a short administration time (15 minutes a day for ten days; Goedert et al., 2015) which would reduce pressure on finite medical resources. Previous research has demonstrated that PA is effective in improving symptoms of neglect, and in some cases, these improvements have extended to activities of daily living (ADLs; Li et al., 2021; Serino et al., 2009; Spaccavento et al., 2016). However, there are limitations to PA that need to be addressed. For example, there has been a lack of engagement found in previous studies given the repetitive nature of this protocol, and clinical recommendations remain tentative as there have been methodological limitations of previous research (e.g., a lack of standardized protocols; Champod et al., 2018, Longley et al., 2021, Spaccavento et al., 2016). Additionally, not all participants benefit from PA and neglect subtypes due to the heterogeneity of stroke locations (e.g., perceptual neglect vs. premotor neglect) may need to be considered when predicting effectiveness (Barrett et al., 2012).

Addressing these issues that are preventing the clinical application of PA procedures, Champod et al. (2014) developed a home-friendly, game-like PA software, Peg-the-Mole (PTM), that requires minimal supervision, and minimizes conscious self-correcting in pointing movements.
The procedure requires participants to touch as quickly as possible a target bull's eye held by a cartoon mole presented on an iPad while wearing prism goggles. This protocol uses an algorithm that gradually reduces the time the bull's eye target is available for the pointing response, thus, emphasizing implicit spatial realignment

processes that enhance the after-effects. Additionally, PTM was designed as a game-like procedure to enhance participant engagement, despite the repetitive nature of the task. Previous research by Champod et al. (2014) tested PTM in a sample of healthy young adults and compared the procedure to a typical PA procedure. They found that prism exposure using 15-degree rightward deviating goggles led to adaptation and significant after-effects that were of equivalent magnitude to those of a typical PA procedure. Importantly, PTM was reported to be more enjoyable and engaging than the typical PA procedure (Champod et al., 2014). More recently, our lab completed a pilot study examining the feasibility of implementing a desktop PTM protocol with participants admitted post-stroke at the Nova Scotia Rehabilitation and Arthritis Centre. PTM was administered daily for 10 sessions by occupational therapists over two weeks. Overall, the protocol was found to be feasible and engaging, however, many challenges arose with the desktop version of PTM. Specifically, the desktop version was problematic for wheelchair users as the desk could not be easily moved around and many could not reach, and this treatment could not be offered to participants at beside. Therefore, we propose to address these challenges by testing a PTM app administered on an iPad, which will allow more flexibility for administration and accessibility.

To further progress towards the clinical implementation of PTM, we plan to investigate the effectiveness and feasibility of implementing our new iPad PA procedure to the inpatient and outpatient environments. If successful, the proposed treatment will represent significant progress towards the clinical implementation of PA by addressing important treatment barriers. Ultimately, this treatment will provide access to home treatment, and decrease cost, travel, and stress on the healthcare system. We will determine who is likely to benefit from this treatment and will aid in achieving more accessible continuing care and ameliorating the well-being of stroke participants by improving rehabilitation outcomes.

PARTICIPANTS

Individuals who have experienced a right-hemisphere stroke will be recruited from the Acquired Brain Injury Program of the Nova Scotia Rehabilitation and Arthritis Centre (NSRAC), the Early Supported Stroke Discharge Team (ESD), the Acute Stroke Program of the Valley Regional Hospital (VRH), and from the community through various support groups (e.g., Colchester Stroke Club, Heart and Stroke Foundation, etc.). All participants enrolled in the study will undergo a baseline assessment. If it is determined from the baseline assessment that the participant is currently experiencing symptoms of neglect (i.e., showing impaired performance on at least 2 subtests of the Behavioural Inattention Test (BIT) and/or at least two ratings of "often" on the Halifax Neglect Impact Scale Caregiver Version (filled out by a caregiver and/or clinician)), the participant will continue to be enrolled in the clinical trial and will complete all remaining aspects of the study (i.e., two weeks of training on PTM and two more assessments). Eligible participants with neglect will either undergo the inpatient or outpatient PTM treatment protocol depending on their predicted length of hospital stay. A hybrid model will be adopted (i.e., integration of the inpatient and outpatient protocol) if a participant is discharged before treatment is completed.

If it is determined that the participant is not currently experiencing symptoms of neglect based on their performance on the baseline assessment, they will not be enrolled in the clinical trial beyond the baseline assessment. If interested, all participants will be provided with a summary of standardized test results upon exiting the study. Additionally, the summary of results may be provided to the participant's clinician if consent is provided by the participant.

Sample Size

G*Power [http://www.gpower.hhu.de] was used to calculate the appropriate sample size for the present study. Based on a review of the effectiveness of prism adaptation (Goedert et al., 2015) we expected a large effect size on neglect severity measures. With an a-level of .05 and 80% power, 15 participants with neglect per group would be the minimum sample size comparing two groups between three time points. Therefore, we planned a sample size of 20 in each group to account for attrition. It is expected that 40 adults with spatial neglect will be recruited for the present study. In order to achieve this number with our current screening protocols, it will likely be necessary to test up to an additional 40 participants with right hemisphere stroke.

Inclusion and Exclusion Criteria

Individuals with right hemisphere stroke will be recruited through the ESD, the Acquired Brain Injury Program of the NSRAC, the Acute Stroke Program of the VRH, and from various stroke support groups in the community. To be eligible to participate in the baseline assessment of the study, participants must: 1) have experienced a right hemisphere stroke; 2) be willing and able to hear about the study and consent to participate; 3) be medically stable; 4) have self-reported normal to corrected-to-normal visual acuity 5) be able to point with the unaffected limb; and 6) live within a two hour radius of Halifax. Exclusion criteria: other existing neurological disorders (e.g., dementia, multiple sclerosis, epilepsy). Participants must live within a 2-hour radius from Halifax due to availability of resources and the location of team members. We will have assessors and testers located in both Halifax and Wolfville. The testers and assessors will be required to commute to the participants' homes and/or the inpatient units. Therefore, to ensure we are able to commute these distances and meet demands, we need to have a limit as to how far the participants will be located in proximity to our testers and assessors.

To be included in the clinical trial beyond the baseline assessment, participants must meet the inclusion/exclusion criteria listed above as well as experience current symptoms of neglect as defined by impaired performance on at least 2 of the BIT subtests and/or at least two ratings of "often" on the Caregiver version of the Halifax Neglect Impact Scale (to be filled out by caregiver and/or clinician).

RESEARCH PLAN

Study design

The current study will use a longitudinal, double-blind, quasi-randomized controlled design. Once it has been determined from the baseline assessment that a participant is currently experiencing neglect symptoms and is therefore eligible to participate in the entire clinical trial, there will be two conditions into which the participant could be randomized. The first will be the experimental condition which will involve using PTM with 15-degree rightward deviating goggles. The selection of the 15-degree deviating prism goggles was based on previous research by Hagen (2018) and McKenzie (2018) which indicated that 15-degree deviating goggles produce significant after-effects that translate to functional tasks (e.g., throwing and aiming). Our second condition (active control) will involve using PTM with 5-degree rightward deviating goggles. Typically, clear goggles (sham) are used as a control. However, a complete absence of optical shift will indicate to participants that they have not been assigned to the treatment condition. Therefore, the use of 5-degree deviating goggles will maintain the double-blind methodology. Previous research has found a significant difference in direct effects and after-effects when comparing 5-degree and 15-degree deviating goggles, with larger strength of visual shift (i.e., 15 degrees) leading to a larger magnitude of prism adaptation effects (Hagen, 2018; McKenzie, 2018). Group assignment will be done with masked envelopes and assessors will be blind to treatment group. Participants will use PTM for five sessions per week during two consecutive weeks. Outcome measures will be

collected immediately before, immediately after, and one-month post-treatment and compared between conditions. After-effects will be measured, and training logs will be completed after each PTM session.

Research Question

The present study will examine whether a new, user-friendly, and accessible clinical protocol (PTM) is feasible and effective as an inpatient and outpatient treatment for spatial neglect after stroke.

Objectives

1. Examine the feasibility of using PTM as a treatment for neglect post-stroke in rehabilitation settings as well as in the home environment of neglect participants.

2. Examine the short- and longer-term effectiveness of using PTM as a treatment for neglect post-stroke in rehabilitation settings as well as in the home environment.

Hypotheses

1. Participants with left-sided neglect who undergo 10 sessions (i.e., two weeks) of prism adaptation training with 15-degree deviating goggles (experimental group) will show larger after-effects and a greater reduction in severity of neglect (as measured by the Behavioural Inattention Test) immediately after training and at one-month follow-up, compared to those who complete training using the 5-degree goggles (active control group).

2. Participants in the experimental group (i.e., 15-degree goggles) will have better functional outcomes (as measured by select tasks from the Behavioural Inattention Test and the Standardised Activities of Daily Living Test) immediately after training and at one-month follow-up, compared to those who complete training using the 5-degree goggles (active control group).

3. The feasibility of implementing this intervention will be examined through treatment compliance (e.g., the proportion of participants that complete the trial), self-reported level of enjoyment, and perceived ease of use of the treatment. The goal will be to determine the extent to which the PTM protocol can be successfully delivered to participants. It is expected that, on average, participants will complete at least 80% of all protocol components (e.g., training, training logs, outcome measures, etc.). Several factors that could negatively impact treatment adherence and effectiveness (e.g., symptoms of depression and fatigue) will also be explored.

Recruitment

If a participant is currently involved or was previously involved with rehabilitation services at the ESD, NSRAC, or VRH, their attending clinician will be the first to approach them regarding their participation in this research project. Clinicians will ask if they would like their names and phone numbers passed on to the research team for more information (see *Appendix* A for Advertisement). Participants will be asked whether they consent to have their names and phone numbers passed on to the research team, and this consent will be provided verbally. If consent is obtained, then a researcher will approach the participant, and obtain written consent if the participant is interested in participating. For individuals in the community who are part of various stroke support groups, members of the research team will attend stroke group meetings and provide an overview of the study and inclusion/exclusion criteria, if possible. Advertisements will also be circulated. If interested in

participating, individuals may approach the research team (in person, by phone, by email) and the research team will obtain written consent privately with the participant before study participation.

Intervention

Prism adaptation will be administered using a computer pointing task called PTM to present pointing targets, record pointing locations, and provide accuracy feedback. The procedure requires participants to touch as quickly as possible a target bull's eye held by a cartoon mole presented on an iPad while wearing either 15-degree rightwards deviating prism goggles (experimental condition) or 5-degree rightwards deviating prism goggles (control condition). Participants will not be informed which goggles they have received. The mole holding the target appears in different locations on the screen. Each time the participant presses the screen, the mole provides visual and auditory feedback on accuracy and reaction time. The PTM software uses an algorithm that adapts the speed at which the targets are presented to the pointing speed of individual participants. Therefore, it monitors the participant's pace and accuracy and controls the rate of presentation of the moles to encourage ballistic movements and to reduce self-correction in pointing movements. More specifically, the algorithm maintains between 50-80% accuracy in performance. The exposure task will last approximately fifteen minutes depending on the speed of responses.

Measures

<u>Screening and Baseline Measures (given before treatment and not repeated)</u>

Health history:

1. Health history questionnaire: a screening questionnaire administered over the phone with the assessor. See *Appendix B* for the health history questionnaire.

2. Stroke Impact Scale – Short Form (SF-SIS; Jenkinson et al., 2013): a modified version of the Stroke Impact Scale. Includes 8 self-report items examining multidimensional stroke outcomes (i.e., strength, activities of daily living, hand function, mobility, communication, emotion, memory and thinking, and participation). See *Appendix C*.

Overall cognitive function:

1. Judgement of Line Orientation (JLO; Benton et al., 1983): a measure of visuospatial judgement and awareness. See *Appendix D* for two example stimuli of the JLO (all 15 items are the same structure with lines in different positions).

2. Confrontation Visual Field Test: A brief visual field exam. See *Appendix E* for scoring sheet.

3. The Montreal Cognitive Assessment (MoCA; Nasreddine et al., 2005): Screening test of overall cognitive abilities. See *Appendix F*.

4. Digit Span (Forward and Backward): A test of short-term memory. See *Appendix G* for scoring sheet.

5. Trail Making Test: A test measuring the ability to search and shift between automatic sequences. See *Appendix H*.

6. Attention-Motor Task (AMT; et al., 2000): a reaching task that characterizes neglect into either a primarily attentional/perceptual deficit or premotor deficit. See *Appendix I* for the instruction sheet.

7. Landmark Task: an abbreviated version of the Landmark Task presented in Bisiach et al. (1998). The task is used to classify neglect into a primarily attentional/perceptual deficit or premotor deficit. See *Appendix J* for an example stimulus page of the Landmark task (each stimulus page is the same structure with different black and red line lengths).

<u>Outcome Measures</u>

Tests assessing neglect:

1. Behavioural Inattention Test (BIT; Wilson et al., 1987): a battery of nine tests to assess the presence and severity of visual neglect. All conventional subtests will be administered. See *Appendix K* for BIT stimuli.

2. After-effects from PTM memory-guided pointing task: A modified proprioceptive-and-visual straight-ahead outcome task without the use of an occlusion board will be performed immediately before and after each training session of PTM. Accuracy will correspond to the average horizontal distance (for pre-PTM and post-PTM in each session), in degrees of visual angle, between the participant's end point and the target bull's eye. The magnitude of after-effects will correspond to the shift in pointing performance in degrees of visual angle between pre- and post-PTM performance.

Tests assessing impact of neglect on activities of daily living:

1. Behavioural Inattention Test (BIT; Wilson et al., 1987): subtests to assess the impact of visual neglect on functional tasks. Only three behavioural subtests will be performed: Article Reading, Menu Reading, and Picture Scanning. See *Appendix K* for BIT stimuli.

2. Eschenbeck Standardized Activities of Daily Living (SADL; Eschenbeck et al., 2010): an assessment for behavioural changes due to neglect. One subtest will be performed: Filling Out a Form. See *Appendix L*. It should be noted that all identifying information is removed from the form once scoring is completed (i.e., before storing the form with study data).

3. Halifax Neglect Impact Scale (Caregiver and Patient versions): an in-house task used previously in neglect participants. Measures participant awareness of symptoms compared to caregiver. See *Appendix M and Appendix N* for the Caregiver and Patient versions of the Halifax Neglect Impact Scale, respectively.

Participant Feedback and Feasibility

1. Exit Questionnaire including questions from a modified Intrinsic Motivation Inventory (Ryan & Deci, 2000). See *Appendix O* for the exit questionnaire.

2. Training logs: tracking participant's compliance with the intervention schedule daily. To be completed by the participant after each training session. See *Appendix P* for a training log.

Tests assessing mental health:

1. Hospital Anxiety and Depression Scale (HADS; Zigmond & Snaith, 1983): a self-report measure used to detect states of depression and anxiety in the setting of inpatient or outpatient clinics. See *Appendix Q*.

2. Fatigue Severity Scale (FSS; Krupp et al., 1989): a self-report questionnaire that measures the severity of fatigue and its effect on activities. See *Appendix R* for the FSS.

Although adverse events are not expected, any that occur will be recorded for safety purposes.

The member of the research team that conducts a participant's assessments will not be the same member of the research team that trains the participant on the intervention. This procedure is to ensure that the assessor is blind to the group (i.e., experimental/control) of the participant they are assessing. Refer to *Appendix S* for task descriptions, durations, and the administration timeline.

PROCEDURE

Outpatient

If a participant is currently enrolled in services provided by the ESD, NSRAC, or VRH, or has previously been enrolled in such services, attending stroke clinicians will be the first to approach the participants regarding participation in the research project. Participants interested in participating in the study will verbally consent to have their names and phone numbers forwarded to the research team. A member of the research team will call each participant to discuss and obtain informed consent. If participants are members of a stroke support group within the community, research team members will circulate study advertisements and will visit the groups to provide an overview of the study, if possible. If interested and eligible, individuals may contact the research team to express interest and complete the consent process over the phone or in person. An electronic consent form will be provided to each participant via REDCap (Research Electronic Data Capture). A health history self-report questionnaire and the Short Form of the Stroke Impact Scale (SF-SIS; Jenkinson et al., 2013) will then be administered online with guidance from the research team member. The health history form will verify inclusion/exclusion criteria.

If participants do not have access to the technology necessary to complete electronic questionnaires and view an electronic consent form, a research team member will travel to the participant (inpatient or at home) and provide them with guidance and an iPad to complete the electronic consent form and the health history questionnaire and the SF-SIS. If a participant wishes to have a paper copy of the consent form to keep, this will be provided. To ensure research staff safety, before completing home visits, the Assessor will send their assigned participant the "Home visit safety checklist" (a modified version of the safety checklist used by NSH employees through the ESD; *Appendix T*) using REDCap and ask that they fill it out before their scheduled appointment. Any required follow-up of the safety checklist or safety planning will be completed over the phone with the participant's Assessor.

Once initial screening is complete and it is determined that an individual is eligible to participate in the baseline assessment, a blinded Assessor will visit the participant to complete the baseline assessment tasks. If the participant prefers to travel to one of our secure and private laboratories (i.e., Dr. Eskes' laboratory in Halifax located at Dalhousie, or, Dr. Champod's laboratory located at Acadia), to complete the baseline assessment, they will be placed in a private testing room with the research staff completing the assessment. Scores on the baseline assessment tasks will be used to determine whether participants are eligible to continue to be enrolled in the clinical trial. Specifically, participants who are not currently experiencing symptoms of neglect as determined by the scores on the baseline assessment tasks (i.e., do not have at least two scores showing impaired performance on the BIT and at least 2 ratings of "often" on the Halifax Neglect Impact Scale Caregiver version filled out by a caregiver and/or clinician) will be informed that they are not eligible to continue participation in the study and will be provided with a standardized tests results summary form of the baseline assessment upon exit (see *Appendix U*).

If it is determined that the participant is currently experiencing symptoms of neglect as defined by at least two scores showing impaired performance on the BIT and/or at least 2 ratings of "often" on the Halifax Neglect Impact Scale Caregiver version filled out by a caregiver and/or clinician), they will receive three more visits (i.e., one training visit and two assessment visits). Participants who will continue to be enrolled in the clinical trial following the baseline assessment will be assigned a Trainer who is not blind to treatment condition and will meet with the same blinded Assessor following treatment.

Following this baseline home visit, the Trainer will return for home visit number two. Participants will be randomized, with concealed allocation, to either the control condition or the experimental condition, and randomization will be stratified by neglect severity which will be measured using the Behavioural Inattention Test (BIT; Wilson et al., 1987) and the Halifax Neglect Impact Scale (Caregiver version). Specifically, participants will be considered as having "mild" neglect if they fail less than 3 subtests on the BIT, or when only functional symptoms are reported. Participants will be considered as having "moderate to severe" neglect when they fail 3 or more subtests on the BIT. We will be using "covariate-adaptive randomization" as cited in Sella et al. (2021) to randomize participants. In lay terms, this randomization mechanism works as follows: with two groups, you can randomly assign your first two participants to the two groups (i.e., either A-B or B-A). For participant 3, an algorithm calculates what would happen if that participant were added to each group, and then chooses the assignment based on the option that minimizes the variation in your target variable. Participant 4 automatically will be assigned to the other group to keep the groups balanced. Then, participant 5 gets balanced, and this pattern of random assignment continues for all other participants. The Trainer will bring the sanitized equipment (i.e., iPad with keyboard and installed PTM app, prism goggles) for the treatment sessions that will take place during the following two weeks. The Trainer will provide an in-person introduction to the use of the PTM software. They will ensure that the proper treatment set-up is in place and will administer practice PTM trials so that the participant becomes familiar with the software. If the participant has a caregiver available, they may choose to be present during the initial training session of PTM in case the participant needs help regarding instructions or set-up at a later training session. The participant and caregiver will have an opportunity to ask questions or clarify anything that is unclear. The participant will be provided with a daily game instructions sheet and checklist (*Appendix W),* as well as a "Connecting Bluetooth Keyboard and iPad instructions" sheet that can be used during training (*Appendix X*). The Trainer will then observe the first treatment session to confirm that the correct procedure is used. The Trainer will help the participant to create a schedule for the remaining nine sessions and the participant will be asked if they prefer to be sent daily emails or text reminders for their therapy sessions. Participants will then complete ten treatment sessions within two

weeks where they will be required to use PTM for 15 minutes a day and test themselves for after-effects using the same software.

Within one week after the last treatment session, the Assessor will return for the third visit. If the participant prefers to travel to one of our secure and private laboratories to complete the immediate follow-up assessment, they will be asked to bring their training equipment to the visit and will be placed in a private testing room with the research staff completing the assessment. The assessor will first ask the participant to set up and complete a PTM training session independently. The assessor will observe a complete training session (including pre- and post-games and PTM) to confirm that the correct procedure has been used by the participant throughout training. The assessor will record the steps of the protocol that are completed by the participant using the "Peg-the-Mole Gameplay Observation Checklist" to measure adherence to the protocol (*Appendix Y*). Once the gameplay observation is complete, the assessor will administer the outcome assessments, including the exit questionnaire. This exit questionnaire will include standardized questions based on the modified Intrinsic Motivation Inventory (Ryan & Deci, 2000). The questionnaire will give participants the opportunity to provide feedback. The exit questionnaire interview, the Halifax Neglect Impact Scale (caregiver and patient versions), as well as select tests (i.e., picture scanning, menu reading, and article reading on the BIT) will be recorded using an audio recorder that will be locked in a filing cabinet in Dr. Eskes or Dr. Champod's secure laboratory. Responses will later be transcribed for analyses, and the recording will be destroyed.

The assessor will return within 4-6 weeks of the last training session to complete the final follow-up assessment (visit #4). If the participant prefers to travel to one of our secure and private laboratories to complete the one-month follow-up assessment, they will be placed in a private testing room with the research staff completing the assessment. The Assessor will administer the outcome assessments, including all conventional subtests and three behavioural subtests of the BIT (Article Reading, Menu Reading, and Picture Scanning), one subtest of the Eschenbeck SADL (Filling Out a Form), the Halifax Neglect Impact Scale (patient version), the FSS and the HADs. If the participant has a caregiver present, the caregiver will be asked to fill out the Halifax Neglect Impact Scale (caregiver version) during baseline and outcome assessments. The responses to the Halifax Neglect Impact Scale (patient and caregiver versions) as well as select tests (i.e., picture scanning, menu reading, and article reading on the BIT) will be recorded using an audio recorder that will be locked in a filing cabinet in Dr. Eskes or Dr. Champod's secure laboratory. Responses will later be transcribed for analyses, and the recordings will be destroyed. If a participant decides to discontinue the study early, the participant will continue to receive their current care plan, as our protocol is being given in addition to any other usual treatments. All data collected up to the date the participant withdraws will be maintained in the study records to be included in the study-related data analyses. It should be noted that an additional research team member may be present at screening, all assessment visits, and the training session for safety purposes and to aid with testing. Upon exit of the study, participants who completed the entire clinical trial will receive standardized tests results summary forms outlining scores on the baseline assessment (*Appendix U)* and scores on the one-month follow-up assessment *(Appendix V*), if interested.

Inpatient

The same procedure as the outpatient group will be used, but the PTM treatment will instead be used by the participant at bedside. The inpatient screening, baseline, and outcome assessments will be conducted by the Assessor in a quiet room. See below for a study timeline.

Community recruitment:
Study staff will visit stroke support groups within the community (if possible) and provide an overview of the study. Advertisements with study information will be circulated. If interested and eligible, individuals may contact study staff.

Clinician referrals:
Clinicians will identify patients with right hemisphere stroke who meet eligibility criteria and will refer to the study if verbal consent is provided by the patient.

Consent & Initial Screening:
Consent is reviewed on the phone or in person with the participant. A health history form and Stroke Impact Scale Short Form are also completed. The health history form will confirm inclusion/exclusion criteria for participating in the Baseline

Baseline Assessment (second screen):
All eligible participants complete a 2-hour assessment including tests of general cognitive functioning, mood, and neglect symptoms.

Ineligible participants:

Participant does not show impaired performance on at least 2 of the BIT subtests and does not receive at least 2 ratings of "often" on the Caregiver version of the Halifax Neglect Impact Scale

Study participation will end for participants who do not currently experience symptoms of neglect as determined by Baseline Assessment results. A summary of baseline standardized results will be provided upon exiting the study.

Eligible participants:

Participant does show impaired performance on at least 2 of the BIT subtests and/or receives at least 2 ratings of "often" on the Caregiver version of the Halifax Neglect Impact Scale

Study participation will continue for participants who are currently experiencing symptoms of neglect, based on Baseline Assessment results. Participants will be assigned a Trainer and will meet with the same Blinded Assessor post-treatment.

Clinical Trial:
Participants will be assigned to one of two conditions. They will complete 10 days of training over two weeks. Post-training assessments will be completed within one-week and one-month following the last day of training. A summary of standardized results will be provided upon exiting the study.

EQUIPMENT

Each participant enrolled in the clinical trial who completes training on PTM will be supplied with a pair of Welder goggles with Fresnel prisms inducing a rightwards optical shift of either 5 degrees or 15 degrees (Insight Optometry Group, Halifax, Canada) to be used during PTM. The PTM software will be administered using a case-protected 9th generation Apple iPad. A wireless QWERTY computer keyboard will be placed directly in front of the participant to align the participant with the center of the tablet. PTM and REDCap applications will both be downloaded and available on the iPad (i.e., to complete PTM training and training logs). The AMT and the Landmark Task will be administered on a Microsoft Surface Pro Tablet. Data from PTM, AMT, and Landmark Task will be stored on a secure Dalhousie-managed website. The server is located in a controlled area in the Killam library on Dalhousie campus and data backup and recovery is managed by Dalhousie. User access is restricted by username/password security and access is given to approved researchers by site administrator. Access levels are designated to specific studies with researchers limited to their own studies.

Upon completion of training, the assessor will collect all equipment from the participant. All equipment will be properly sanitized between participants.

ANALYSIS OF DATA

For the analysis of effectiveness, we will examine direct effects (i.e., errors in degrees of visual angle during PTM), immediate after-effects, and the effect of PTM on severity of neglect symptoms immediately and at one-month post-treatment. To analyze direct effects of the prism goggles, a mixed ANOVA will be used with Condition (15- and 5-degree) and Week (1 and 2) as independent variables and the mean magnitude of pointing errors in degrees of visual angle during the first 10 trials of PTM as the dependent variable. After-effects will be examined using a mixed ANOVA with Condition as a between-subjects factor and Week as a within-subjects factor. Mean pointing errors in degrees of visual angle during the outcome pointing task will be the dependent variable. Mixed ANOVAs will also be used to examine the effect of PTM on neglect severity and activities of daily living. Specifically, Condition will be a between-subjects factor and Time (Pre-PTM, Immediate Post-PTM, 1-month Post-PTM) will be a within-subjects factor. The total score of the BIT, the scores of each separate subtest within the BIT and SADL, as well as the percentage of participants who fail each subtest before and after will be used as dependent variables.

For feasibility, we will compute descriptive statistics and examine mean ratings derived from participant exit interviews. Additionally, upon completion of training, the number of completed PTM training sessions, daily training logs, and outcome assessments will be tallied for each participant and the proportion of completion of each PTM component will be calculated. For example, if a participant completes three daily training logs out of the five daily logs assigned, their proportion of completion for the daily training log component will be 3/5 = 0.6 (or 60%). The protocol will be deemed feasible if the mean proportion of completion in the overall sample is at least 80% for all protocol components. Feedback on the Exit Questionnaire and the Halifax Neglect Impact Scale (Patient and Caregiver versions) will be transcribed and analyzed using Thematic Analysis. Microsoft Word and NVivo software offered through Dalhousie University licensing will be used to transcribe and analyze qualitative data.

Exploratory analyses will be used to examine factors that may influence treatment outcomes (e.g., depression, anxiety). Demographic and clinical characteristics (e.g., age, neglect severity) will be explored using ANOVAs.

All test results will be corrected for multiple comparisons.

CONFIDENTIALITY

Procedures will be utilized to ensure personal health information and study data are protected. All study staff will be trained in confidentiality and only those directly involved with this research project will have access to participant data. Participants will be assigned a unique ID code (e.g., P001) to de-identify their study data. Participant ID codes will be used to label all documents, records, and data.

Electronic consent forms, health screening questionnaires, the SF-SIS, training logs and personal contact information will be collected and stored using REDcap Electronic data capture tools, hosted at Nova Scotia Health. REDCap is a secure, web-based application designed to support data capture for research studies, providing: 1) an intuitive interface for validated data entry; 2) audit trails for tracking data manipulation and export procedures; 3) automated export procedures for seamless data downloads to common statistical packages; and 4) procedures for importing data from external sources. Summaries of raw data from the BIT, SADL, JLO, FSS, HADS, Landmark, MoCA, Digit Span, Trails, Halifax Neglect Impact Scales, and the Exit Questionnaire will be collected and inputted into REDcap.

Any other electronic data will be de-identified and stored on password protected computers or saved on Dr. Eskes' lab network that is behind a secure Dalhousie firewall and password protected, with access restricted per staff role. When a participant is no longer active in the study, all electronic data collected outside of REDcap will be relocated to a password protected hard drive in Dr. Champod's secure laboratory in the Acadia Centre for Neuroscience and Cognitive Health. While participants are active in the study, all paper records and audio recordings stored on the audio recorder will be stored in a locked filing cabinet in Dr. Eskes' secure laboratory in the Life Sciences Research Institute (participants in Halifax) or in Dr. Champod's secure laboratory (participants in the Valley). Once audio recordings have been transcribed for analysis, the audio recordings will be destroyed. When participants are no longer active in the study, all paper records will be moved and stored in Dr. Champod's secure laboratory in the Acadia Centre for Neuroscience and Cognitive Health. All data are being collected by Dalhousie/Acadia researchers and therefore there is no data being transferred to Dalhousie/Acadia research from NSH.

HARMS

The training intervention in the present study is a non-invasive, behavioural intervention. We do not foresee any serious adverse events involved with participating in the current study as there are no invasive procedures. PA has been used frequently in stroke participants with neglect symptoms, with no adverse events reported (Fortis et al., 2010; Làdavas et al., 2011; Rode et al., 2015). Potential minor harms may include frustration and mental fatigue. Given that the study involves a behavioural intervention, no medical emergencies are anticipated due to participation. However, participants will be informed that they may withdraw at any time without penalty, and this will not interfere with any other ongoing treatments. All participants will receive phone calls during their training from the trainer who will monitor and document any adverse events. Inpatients and outpatients will each continue their standard of care for stroke rehabilitation as to the inpatient or outpatient protocol.

BENEFITS

This present study uses an innovative protocol that can provide access to home treatment for

individuals with spatial neglect. The PTM procedure has the potential to improve participant recovery and quality of life due to its features that promote treatment availability and adherence. The results will help us determine if the treatment can be used successfully in inpatient and outpatient settings and who is likely to benefit. Ultimately, these findings should aid in achieving improved continuing care, more accessible health services, and amelioration of the well-being of Nova Scotians by improving rehabilitation outcomes, independence, and quality of life after stroke.

DISCLOSURE OF COMPENSATION OR BENEFITS

Participants will receive up to $50 compensation for their participation in this research study as a small "thank you" for their time, depending on their eligibility. Regular payments will be made throughout the protocol. Participants who participate in the Baseline Assessment will receive $20. If eligible to participate in the clinical trial, participants will receive $15 for the Post-treatment Assessment and $15 for the Follow-up Assessment.

The investigators have a potential financial interest in this study. The results of this study may be used to develop a tool for improving neglect symptoms that could be commercialized in the future. If this tool is developed commercially in the future, profits may be available to the researchers and institution. This potential benefit is noted in the consent form.

KNOWLEDGE TRANSLATION PLAN

Our team will engage in various knowledge translation activities (e.g., via the presentation of the results in media outlets, stroke support groups [e.g., Colchester Stroke Club], clinical rounds at the NSRAC and VRH). We also plan to publish in a top peer-reviewed journal in the field of rehabilitation such as Archives of Physical Medicine and Rehabilitation Medicine. We plan to present the findings at conferences reaching a broad range of audiences such as the American Congress of Rehabilitation Medicine and the Canadian or World Stroke Conference that are attended by both researchers and clinicians.

References

Appelros, P., Karlsson, G. M., Seiger, A., & Nydevik, I. (2003). Prognosis for participants with neglect and anosognosia with special reference to cognitive impairment. *Journal of rehabilitation medicine*, *35*, 254–258. https://doi.org/10.1080/16501970310012455

Barrett, A. M., Goedert, K. M., & Basso, J. C. (2012). Prism adaptation for spatial neglect after stroke: translational practice gaps. *Nature reviews. Neurology*, *8*, 567–577. https://doi.org/10.1038/nrneurol.2012.170

Basagni, B., Errante, A., Pinardi, C., De Gaetano, K., Crisi, G., De Tanti, A., & Fogassi, L. (2019). Rehabilitation of unilateral spatial neglect: A combined behavioral and fMRI single-case study. *Neuropsychology, 33*, 343–357. https://doi.org/10.1037/neu0000523

Benton, A. L., Hamsher, K., Varney, N. R., & Spreen, O. (1983). *Contributions to neuropsychological assessment*. New York: Oxford University Press.

Bisiach, E., Ricci, R., Lualdi, M., & Colombo, M. R. (1998). Perceptual and response bias in unilateral neglect: two modified versions of the milner landmark task. *Brain and cognition*, *37*, 369–386. https://doi.org/10.1006/brcg.1998.1003

Champod, A. S., Frank, R. C., Taylor, K., & Eskes, G. A. (2018). The effects of prism adaptation on daily life activities in participants with visuospatial neglect: a systematic review. *Neuropsychological rehabilitation*, *28*, 491–514. https://doi.org/10.1080/09602011.2016.1182032

Champod, A. S., Taylor, K., & Eskes, G. A. (2014). Development of a new computerized prism adaptation procedure for visuo-spatial neglect. *Journal of neuroscience methods*, *235*, 65–75. https://doi.org/10.1016/j.jneumeth.2014.05.023

Chen, P., Chen, C. C., Hreha, K., Goedert, K. M., & Barrett, A. M. (2015). Kessler Foundation Neglect Assessment Process uniquely measures spatial neglect during ADLs. *Archives of physical medicine and rehabilitation*, *96*, 869–876.e1. https://doi.org/10.1016/j.apmr.2014.10.023

Dai, C. Y., Liu, W. M., Chen, S. W., Yang, C. A., Tung, Y. C., Chou, L. W., & Lin, L. C. (2014). Anosognosia, neglect and quality of life of right hemisphere stroke survivors. *European journal of neurology*, *21*, 797–801. https://doi.org/10.1111/ene.12413

Dalmaijer, E. S., Van der Stigchel, S., Nijboer, T., Cornelissen, T., & Husain, M. (2015). CancellationTools: All-in-one software for administration and analysis of cancellation tasks. *Behavior research methods*, *47*, 1065–1075. https://doi.org/10.3758/s13428-014-0522-7

Eschenbeck, P., Vossel, S., Weiss, P. H., Saliger, J., Karbe, H., & Fink, G. R. (2010). Testing for neglect in right-hemispheric stroke participants using a new assessment battery based upon standardized activities of daily living (ADL). *Neuropsychologia*, *48*, 3488–3496. 10.1016/j.neuropsychologia.2010.07.034

Fortis, P., Maravita, A., Gallucci, M., Ronchi, R., Grassi, E., Senna, I., Olgiati, E., Perucca, L., Banco, E., Posteraro, L., Tesio, L., & Vallar, G. (2010). Rehabilitating participants with left spatial neglect by prism exposure during a visuomotor activity. *Neuropsychology*, *24*, 681–697. https://doi.org/10.1037/a0019476

Goedert, K. M., Zhang, J. Y., & Barrett, A. M. (2015). Prism adaptation and spatial neglect: the need for dose-finding studies. *Frontiers in human neuroscience*, *9*, 243. https://doi.org/10.3389/fnhum.2015.00243

Grattan, E. S., Skidmore, E. R., & Woodbury, M. L. (2018). Examining Anosognosia of Neglect. *OTJR: Occupation, participation and health*, *38*, 113–120. https://doi.org/10.1177/1539449217747586

Hagen, K. (2018, May). *Modulation of prism adaptation effects using a game-like procedure in healthy young adults* [Unpublished bachelor's thesis]. Acadia University, Wolfville, NS.

Husain, M., Mattingley, J. B., Rorden, C., Kennard, C., & Driver, J. (2000). Distinguishing sensory and motor biases in parietal and frontal neglect. *Brain: A journal of neurology*, *123*, 1643–1659. https://doi.org/10.1093/brain/123.8.1643

Jenkinson, C., Fitzpatrick, R., Crocker, H., & Peters, M. (2013). The Stroke Impact Scale: validation in a UK setting and development of a SIS short form and SIS index. *Stroke*, 44, 2532-5.

Krupp, L. B., LaRocca, N. G., Muir-Nash, J., & Steinberg, A. D. (1989). The fatigue severity scale. Application to patients with multiple sclerosis and systemic lupus erythematosus. *Archives of neurology*, *46*(10), 1121–1123. https://doi.org/10.1001/archneur.1989.00520460115022

Li, K., & Malhotra, P. A. (2015). Spatial neglect. *Practical neurology*, *15*, 333–339. https://doi.org/10.1136/practneurol-2015-001115

Li, J., Li, L., Yang, Y., & Chen, S. (2021). Effects of Prism Adaptation for Unilateral Spatial Neglect After Stroke: A Systematic Review and Meta-Analysis. *American journal of physical medicine & rehabilitation*, *100*, 584–591. https://doi.org/10.1097/PHM.0000000000001598

Làdavas, E., Bonifazi, S., Catena, L., and Serino, A. (2011). Neglect rehabilitation by prism adaptation: Different procedures have different impacts. *Neuropsychologia, 49*, 1136-1145. https://doi.org/10.1016/j.neuropsychologia.2011.01.044

Langer, K. G., Piechowski-Jozwiak, B., & Bogousslavsky, J. (2019). Hemineglect and Attentional Dysfunction. *Frontiers of neurology and neuroscience*, *44*, 89–99. https://doi.org/10.1159/000494956

Longley, V., Hazelton, C., Heal, C., Pollock, A., Woodward-Nutt, K., Mitchell, C., Pobric, G., Vail, A., & Bowen, A. (2021). Non-pharmacological interventions for spatial neglect or inattention following stroke and other non-progressive brain injury. *The Cochrane database of systematic reviews*, *7*, CD003586. https://doi.org/10.1002/14651858.CD003586.pub4

Mark V. W. (2003). Acute versus chronic functional aspects of unilateral spatial neglect. *Frontiers in bioscience; A journal and virtual library*, *8*, e172–e189. https://doi.org/10.2741/973

McKenzie, L. (2018). *Prismatic strength and the modulation of after-effects on a functional task in prism adaptation* [Unpublished bachelor's thesis]. Acadia University, Wolfville, Nova Scotia.

Mizuno, K., Tsuji, T., Takebayashi, T., Fujiwara, T., Hase, K., & Liu, M. (2011). Prism adaptation therapy enhances rehabilitation of stroke participants with unilateral spatial neglect: a randomized, controlled trial. *Neurorehabilitation and neural repair*, *25*, 711–720. https://doi.org/10.1177/1545968311407516

Nasreddine, Z. S., Phillips, N. A., Bédirian, V., Charbonneau, S., Whitehead, V., Collin, I., Cummings, J. L., & Chertkow, H. (2005). *Montreal Cognitive Assessment (MoCA)* [Database record]. APA PsycTests.

https://doi.org/10.1037/t27279-000

Nys, G. M., van Zandvoort, M. J., van der Worp, H. B., de Haan, E. H., de Kort, P. L., Jansen, B. P., & Kappelle, L. J. (2006). Early cognitive impairment predicts long-term depressive symptoms and quality of life after stroke. *Journal of the neurological sciences*, *247*, 149–156. https://doi.org/10.1016/j.jns.2006.04.005

Ogourtsova, T., Souza Silva, W., Archambault, P. S., & Lamontagne, A. (2017). Virtual reality treatment and assessments for post-stroke unilateral spatial neglect: A systematic literature review. *Neuropsychological rehabilitation*, *27*, 409–454. https://doi.org/10.1080/09602011.2015.1113187

Reitan, R.M. (1958). Validity of the Trail Making Test as an Indicator of Organic Brain Damage. *Perceptual and Motor Skills, 8*, 271-276. https://doi.org/10.2466/pms.1958.8.3.271

Riestra, A. R., & Barrett, A. M. (2013). Rehabilitation of spatial neglect. *Handbook of clinical neurology*, *110*, 347–355. https://doi.org/10.1016/B978-0-444-52901-5.00029-0

Rode, G., Lacour, S., Jacquin-Courtois, S., Pisella, L., Michel, C., Revol, P., Alahyane, N., Luauté, J., Gallagher, S., Halligan, P., Pélisson, D., & Rossetti, Y. (2015). Long-term sensorimotor and therapeutical effects of a mild regime of prism adaptation in spatial neglect. A double-blind RCT essay. *Annals of physical and rehabilitation medicine*, *58*, 40–53. https://doi.org/10.1016/j.rehab.2014.10.004

Rusconi, M. L., & Carelli, L. (2012). Long-term efficacy of prism adaptation on spatial neglect: preliminary results on different spatial components. *The scientific world journal*, *2012*, 618528. https://doi.org/10.1100/2012/618528

Ryan, R. M., & Deci, E. L. (2000). Self-determination theory and the facilitation of intrinsic motivation, social development, and well-being. *American Psychologist, 55*, 68–78. https://doi.org/10.1037/0003-066X.55.1.68

Sella, F., Raz, G., & Cohen Kadosh, R. (2021). When randomisation is not good enough: Matching groups in intervention studies. *Psychonomic bulletin & review*, *28*, 2085–2093. https://doi.org/10.3758/s13423-021-01970-5

Serino, A., Barbiani, M., Rinaldesi, M. L., & Làdavas, E. (2009). Effectiveness of prism adaptation in neglect rehabilitation: a controlled trial study. *Stroke*, *40*, 1392–1398. https://doi.org/10.1161/STROKEAHA.108.530485

Spaccavento, S., Cellamare, F., Cafforio, E., Loverre, A., & Craca, A. (2016). Efficacy of visual-scanning training and prism adaptation for neglect rehabilitation. *Applied neuropsychology adult*, *23*, 313–321. https://doi.org/10.1080/23279095.2015.1038386

Wilson, B., Cockburn, J., & Halligan, P. (1987). Development of a behavioral test of visuospatial neglect. *Archives of physical medicine and rehabilitation*, *68*, 98–102.

Woodard, J. L., Benedict, R. H., Salthouse, T. A., Toth, J. P., Zgaljardic, D. J., & Hancock, H. E. (1998). Normative data for equivalent, parallel forms of the Judgment of Line Orientation Test. *Journal of clinical and experimental neuropsychology*, *20*, 457–462.

Zigmond, A. S., & Snaith, R. P. (1983). The hospital anxiety and depression scale. *Acta psychiatrica Scandinavica*, *67*, 361–370. https://doi.org/10.1111/j.1600-0447.1983.tb09716.x

APPENDIX A
Advertisement

Volunteers Needed for a Research Study

- ✓ **Have you had a right-hemisphere stroke?**
- ✓ **Are you interested in improving health care by volunteering for clinical research?**

If you said 'YES" to these questions, you may be eligible to participate in a research study. Our clinical study aims to test an online rehabilitation program for attention.

We need volunteers who have normal vision (glasses are okay), are able to point with their good limb, and live within a 2-hour drive of Halifax.

What do you need to do?

We would visit you in the hospital or at home and ask you to complete tasks that look at different thinking abilities like attention. This visit will take around 1.5 hours.

If eligible to continue in the study after the first visit, you will receive 3 more visits from study staff over 2 months. You will play a game that involves pointing to targets for 10 short daily sessions.

You will receive an honorarium of up to $50.

If interested in learning more, please contact **Catrina MacPhee** at **902-494-4033** or **EMPATH@dal.ca**

Thank you in advance for helping with this valuable research!

APPENDIX B

Health History Questionnaire

Code:____________________ Date:________________

Background and Health History

Demographics:

Gender:

Pronouns:

Handedness (ask if switched):

Education (years):

First Language:

Age:

Occupation (or previous occupation if retired):

Medical Information:

What is your general state of health? Excellent Very Good Good Fair Poor

Have you ever been diagnosed or treated for any of the following: If YES: Details (meds and time since diagnosis)

- Head injury with loss of consciousness? YES NO
- Seizures? YES NO
- Multiple Sclerosis? YES NO
- Parkinson's Disease? YES NO
- Huntington's Disease? YES NO
- Stroke? YES NO
- Dominant upper limb pain or injury? YES NO
- Sleep disorders? YES NO
- Diabetes? YES NO
- Thyroid problems? YES NO
- High Blood Pressure? YES NO

- Heart Problems? YES NO
- Surgery? YES NO
- Cancer? YES NO
- Visual Problems? YES NO
- Glasses? If yes, are they present? YES NO
- Trouble Hearing? YES NO
- Hearing aid? If yes, is it present? YES NO
- Colour Blindness? YES NO
- COVID-19? YES NO
- Anxiety? YES NO
- Depression? YES NO

Are there any other medical or psychological conditions that we haven't asked about?

What other medical, physical, or psychological treatments are you currently undergoing?

Have you ever experienced any left-sided symptoms after your stroke (e.g., weakness, sensory changes, vision loss, paralysis, etc.)?

Medications:

APPENDIX E

Confrontation Visual Field Test – Scoring Sheet

Confrontation of Visual Fields

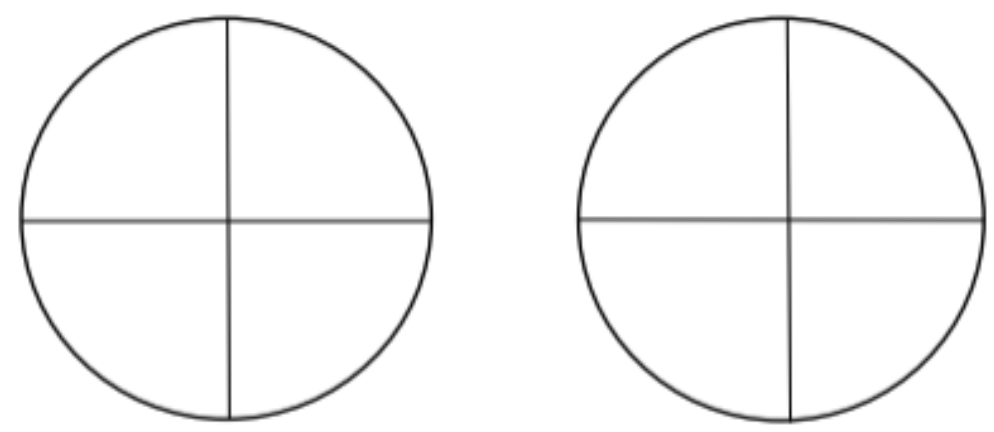

Patient's Left Eye (OS)   Patient's Right Eye (OD)

Visual Field Extinction

Upper:

R	R	L	R	B	L	B	L	B

Lower:

R	R	L	R	B	L	B	L	B

APPENDIX I

Instruction Sheet for the Attention Motor Task

This task is called the Attention Motor task. It helps us know whether differences in attention are related to paying attention or moving your hand to different start positions.
<orient participant to the touchscreen tablet>.<note: participant is to use their RIGHT HAND>

In this task, there are two circles on the screen.

You are going to try to touch one of the circles. You will always start from this green START square *<point to the start square>.*

First, you will see a yellow cross in the centre of the screen *<show the participant the yellow fixation cross>.*
When the cross appears, place your index finger on the green square, but keep looking at the yellow cross. Soon, a snowflake will appear.
Sometimes the snowflake will be green. When a green snowflake appears, release the start button and touch the circle below as <u>quickly</u> as you can. <DEMO>
Sometimes the snowflake will be red. <DEMO> **A red snowflake means 'stop'. If a red snowflake appears, keep your index finger on the START square, and look at the yellow cross.**

Before we begin the task, we'll do some practice. Do you have any questions before we begin?

<the green start square is on the screen and yellow fixation cross appears> **Here is the green START square. Go ahead and press it with your index finger. Keep looking at the yellow cross while you wait for the circle to appear.**

<if the snowflake is green > **Now a green snowflake has appeared. Lift your finger from the green square and touch the circle with the same finger as quickly as you can.**

<if the snowflake is red > **Now the red snowflake has appeared. This means 'stop' – keep your finger on the start square.**

<After they execute the movement> **Good job!**

<practice trials (8 total) >

Good job! Would you like to practice again or are you ready to start the task?

<if redo practice, walk them through it again, if do not want to redo the practice begin the task>

Now let's begin the task. Just to remind you, there are two circles on the screen. While you're waiting for the snowflake to appear, keep looking at the yellow cross. If a green snowflake appears, touch the circle below. If a red snowflake appears, keep your index finger on the START square. Are you ready to begin?

<when the start square changes location at the beginning of the next block>

OK, now your task is the same, but this time you will be starting from this square. <show participant the 'new' start square> *Repeat for each subsequent block as needed.

APPENDIX J

Landmark Task - Example Stimulus

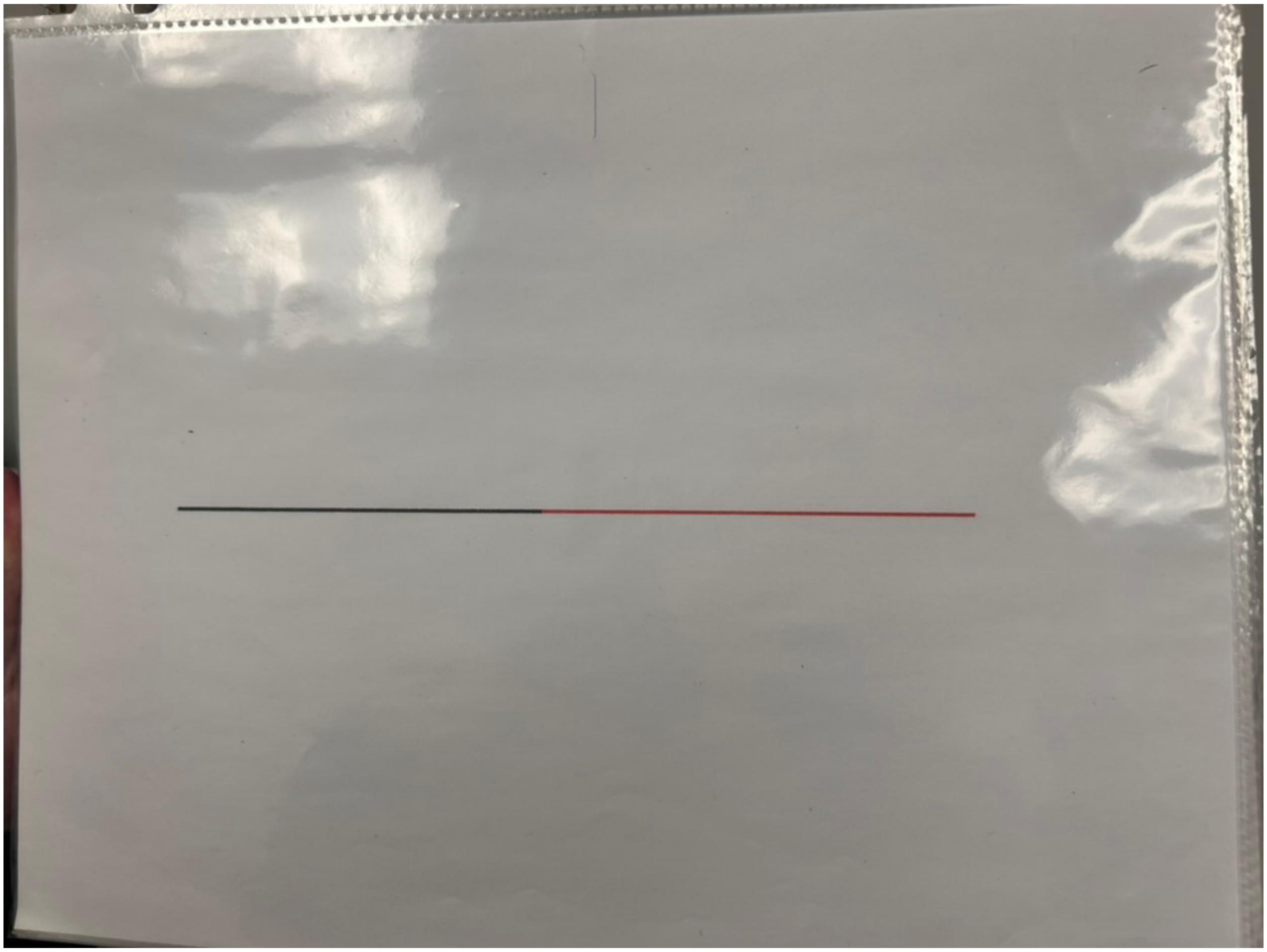

APPENDIX M
Halifax Neglect Impact Scale (Patient version)

Participant ID**:**____________________

Date:____________________________

Halifax Neglect Impact Scale

Self-Rated Patient Version

The following changes can happen to people after a stroke. Please estimate the frequency of each item for you in the <u>last week</u> by circling or marking the best response.

e.g. Never — Rarely — Occasionally — Often — Very Often OR Don't Know ☒

<u>Ambulation & Navigation</u>

1. Bumping into objects/wall on the left while walking or using a wheelchair

Never — Rarely — Occasionally — Often — Very Often | Don't Know ☐

<u>Baseline assessment:</u>
***If item is rated anything higher than "never" ask:**
1. How has this affected your ability to carry out everyday activities?
2. Does this bother you? If yes, how so?

<u>Immediate follow-up assessment:</u>
***If any reported change (improvement or deterioration) from baseline rating, say:**
1. So, before using PTM, your rating was "xx". Now you've said it is "xx". Can you tell me more about this change in rating?

<u>One month follow-up assessment:</u>
***If any reported change (improvement or deterioration) from immediate follow-up rating, ask:**
1. So, when you just finished using PTM, your rating was "xx". Now you've said it is "xx". Can you tell me more about this change in rating?

© 2016 Gail Eskes. Not to be used without permission. Electronic copies may be obtained from gail.eskes@dal.ca
Modified by Anne Sophie Champod and Catrina MacPhee with permission.

Never | Rarely | Occasionally | Often | Very Often | Don't Know

2. Missing left turns while walking or using a wheelchair ☐

Baseline assessment:
***If item is rated anything higher than "never" ask:**
1. How has this affected your ability to carry out everyday activities?
2. Does this bother you? If yes, how so?

Immediate follow-up assessment:
***If any reported change (improvement or deterioration) from baseline rating, say:**
1. So, before using PTM, your rating was "xx". Now you've said it is "xx". Can you tell me more about this change in rating?

One month follow-up assessment:
***If any reported change (improvement or deterioration) from immediate follow-up rating, ask:**
1. So, when you just finished using PTM, your rating was "xx". Now you've said it is "xx". Can you tell me more about this change in rating?

Never | Rarely | Occasionally | Often | Very Often | Don't Know

3. Needing reminders or supervision to move your left foot safely while walking or climbing stairs ☐

Baseline assessment:
***If item is rated anything higher than "never" ask:**
1. How has this affected your ability to carry out everyday activities?
2. Does this bother you? If yes, how so?

Immediate follow-up assessment:
***If any reported change (improvement or deterioration) from baseline rating, say:**
1. So, before using PTM, your rating was "xx". Now you've said it is "xx". Can you tell me more about this change in rating?

One month follow-up assessment:
***If any reported change (improvement or deterioration) from immediate follow-up rating, ask:**
1. So, when you just finished using PTM, your rating was "xx". Now you've said it is "xx". Can you tell me more about this change in rating?

© 2016 Gail Eskes. Not to be used without permission. Electronic copies may be obtained from gail.eskes@dal.ca
Modified by Anne Sophie Champod and Catrina MacPhee with permission.

	Never	Rarely	Occasionally	Often	Very Often	Don't Know
4. Forgetting to lock your left brake, if using a wheelchair						☐

Baseline assessment:
***If item is rated anything higher than "never" ask:**
1. How has this affected your ability to carry out everyday activities?
2. Does this bother you? If yes, how so?

Immediate follow-up assessment:
***If any reported change (improvement or deterioration) from baseline rating, say:**
1. So, before using PTM, your rating was "xx". Now you've said it is "xx". Can you tell me more about this change in rating?

One month follow-up assessment:
***If any reported change (improvement or deterioration) from immediate follow-up rating, ask:**
1. So, when you just finished using PTM, your rating was "xx". Now you've said it is "xx". Can you tell me more about this change in rating?

Scanning in Space

	Never	Rarely	Occasionally	Often	Very Often	Don't Know
5. Missing objects on the left side (e.g., on a shelf, or in the fridge)						☐

Baseline assessment:
***If item is rated anything higher than "never" ask:**
1. How has this affected your ability to carry out everyday activities?
2. Does this bother you? If yes, how so?

Immediate follow-up assessment:
***If any reported change (improvement or deterioration) from baseline rating, say:**
1. So, before using PTM, your rating was "xx". Now you've said it is "xx". Can you tell me more about this change in rating?

One month follow-up assessment:
***If any reported change (improvement or deterioration) from immediate follow-up rating, ask:**
1. So, when you just finished using PTM, your rating was "xx". Now you've said it is "xx". Can you tell me more about this change in rating?

© 2016 Gail Eskes. Not to be used without permission. Electronic copies may be obtained from gail.eskes@dal.ca
Modified by Anne Sophie Champod and Catrina MacPhee with permission.

	Never	Rarely	Occasionally	Often	Very Often	Don't Know
6. Missing items on the left side of your plate						☐

Baseline assessment:
***If item is rated anything higher than "never" ask:**
1. How has this affected your ability to carry out everyday activities?
2. Does this bother you? If yes, how so?

Immediate follow-up assessment:
***If any reported change (improvement or deterioration) from baseline rating, say:**
1. So, before using PTM, your rating was "xx". Now you've said it is "xx". Can you tell me more about this change in rating?

One month follow-up assessment:
***If any reported change (improvement or deterioration) from immediate follow-up rating, ask:**
1. So, when you just finished using PTM, your rating was "xx". Now you've said it is "xx". Can you tell me more about this change in rating?

	Never	Rarely	Occasionally	Often	Very Often	Don't Know
7. Missing left side of sentences or signs when reading						☐

Baseline assessment:
***If item is rated anything higher than "never" ask:**
1. How has this affected your ability to carry out everyday activities?
2. Does this bother you? If yes, how so?

Immediate follow-up assessment:
***If any reported change (improvement or deterioration) from baseline rating, say:**
1. So, before using PTM, your rating was "xx". Now you've said it is "xx". Can you tell me more about this change in rating?

One month follow-up assessment:
***If any reported change (improvement or deterioration) from immediate follow-up rating, ask:**
1. So, when you just finished using PTM, your rating was "xx". Now you've said it is "xx". Can you tell me more about this change in rating?

© 2016 Gail Eskes. Not to be used without permission. Electronic copies may be obtained from gail.eskes@dal.ca
Modified by Anne Sophie Champod and Catrina MacPhee with permission.

<u>Self-Care Issues</u>

8. Needing reminders to wash or shave on the left side of your body

Never — Rarely — Occasionally — Often — Very Often | Don't Know ☐

<u>Baseline assessment:</u>
***If item is rated anything higher than "never" ask:**
1. How has this affected your ability to carry out everyday activities?
2. Does this bother you? If yes, how so?

<u>Immediate follow-up assessment:</u>
***If any reported change (improvement or deterioration) from baseline rating, say:**
1. So, before using PTM, your rating was "xx". Now you've said it is "xx". Can you tell me more about this change in rating?

<u>One month follow-up assessment:</u>
***If any reported change (improvement or deterioration) from immediate follow-up rating, ask:**
1. So, when you just finished using PTM, your rating was "xx". Now you've said it is "xx". Can you tell me more about this change in rating?

9. Having trouble dressing the left side of your body (e.g., finding it difficult to pull up your pants on the left side, or to put your left arm in your sleeve)

Never — Rarely — Occasionally — Often — Very Often | Don't Know ☐

<u>Baseline assessment:</u>
***If item is rated anything higher than "never" ask:**
1. How has this affected your ability to carry out everyday activities?
2. Does this bother you? If yes, how so?

<u>Immediate follow-up assessment:</u>
***If any reported change (improvement or deterioration) from baseline rating, say:**
1. So, before using PTM, your rating was "xx". Now you've said it is "xx". Can you tell me more about this change in rating?

<u>One month follow-up assessment:</u>
***If any reported change (improvement or deterioration) from immediate follow-up rating, ask:**
1. So, when you just finished using PTM, your rating was "xx". Now you've said it is "xx". Can you tell me more about this change in rating?

© 2016 Gail Eskes. Not to be used without permission. Electronic copies may be obtained from gail.eskes@dal.ca
Modified by Anne Sophie Champod and Catrina MacPhee with permission.

10. Needing reminders to keep your left arm in the correct place

Never — Rarely — Occasionally — Often — Very Often | Don't Know ☐

Baseline assessment:
***If item is rated anything higher than "never" ask:**
1. How has this affected your ability to carry out everyday activities?
2. Does this bother you? If yes, how so?

Immediate follow-up assessment:
***If any reported change (improvement or deterioration) from baseline rating, say:**
1. So, before using PTM, your rating was "xx". Now you've said it is "xx". Can you tell me more about this change in rating?

One month follow-up assessment:
***If any reported change (improvement or deterioration) from immediate follow-up rating, ask:**
1. So, when you just finished using PTM, your rating was "xx". Now you've said it is "xx". Can you tell me more about this change in rating?

11. How much of an effect do left-sided symptoms have on your daily functioning?

1	2	3	4	5	6	7	8	9	10
no disabling effect									a severely disabling effect

We've been focusing on some specific areas that may have been affected by left-sided symptoms, but now we want to hear from you about anything else you may have noticed.

BASELINE ASSESSMENT QUESTIONS:

1. What other changes have you noticed since having the stroke?
2. How have these changes impacted everyday life?
3. Do these changes bother you? If yes, how so?

© 2016 Gail Eskes. Not to be used without permission. Electronic copies may be obtained from gail.eskes@dal.ca
Modified by Anne Sophie Champod and Catrina MacPhee with permission.

IMMEDIATE POST-TRAINING ASSESSMENT QUESTIONS:

1. Since you started using the Peg-the-Mole treatment, what changes have you noticed in your left-sided symptoms and in your ability to carry out everyday activities?
2. Since you started using the Peg-the-Mole treatment, what other changes in your daily life have you noticed?

1-MONTH FOLLOW-UP ASSESSMENT QUESTIONS:

1. Since you finished using the Peg-the-Mole treatment, what changes have you noticed in your left-sided symptoms and in your ability to carry out everyday activities?
2. Since you finished using the Peg-the-Mole treatment, what other changes in your daily life have you noticed?
3. Throughout the study, which condition do you believe you were assigned to: the experimental condition or the control condition?

© 2016 Gail Eskes. Not to be used without permission. Electronic copies may be obtained from gail.eskes@dal.ca
Modified by Anne Sophie Champod and Catrina MacPhee with permission.

APPENDIX N

Halifax Neglect Impact Scale (Caregiver Version)

Participant ID:____________________ Relationship to participant: ____________________________

Date:____________________________ Verbal consent obtained?: ____________________________

Halifax Neglect Impact Scale

Caregiver Version

The following changes can happen to people after a stroke. Please estimate the frequency of each item for the participant in the last week by circling or marking the best response.

e.g.

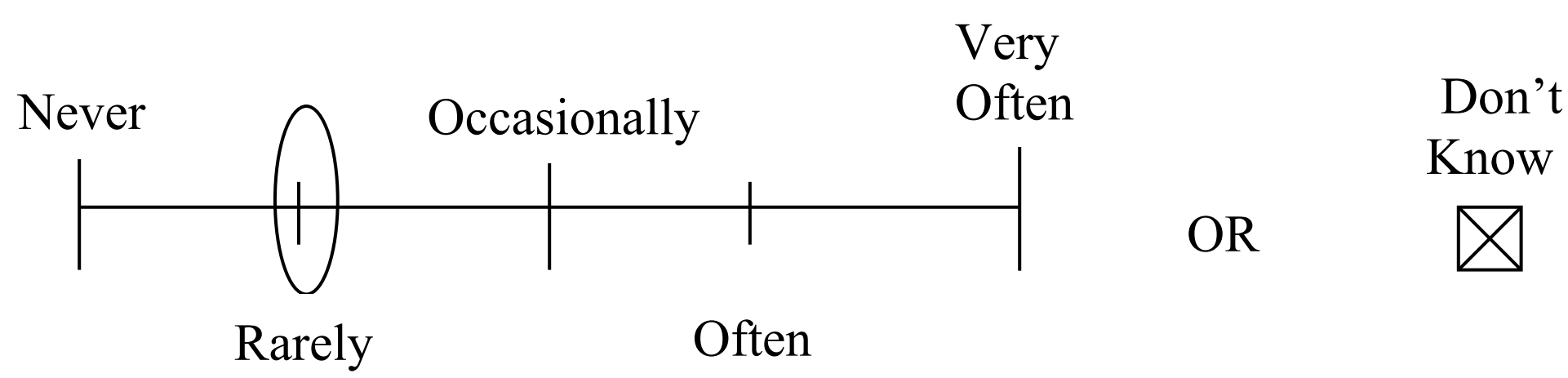

Ambulation & Navigation

1. Bumps into objects/wall on the left while walking or using a wheelchair

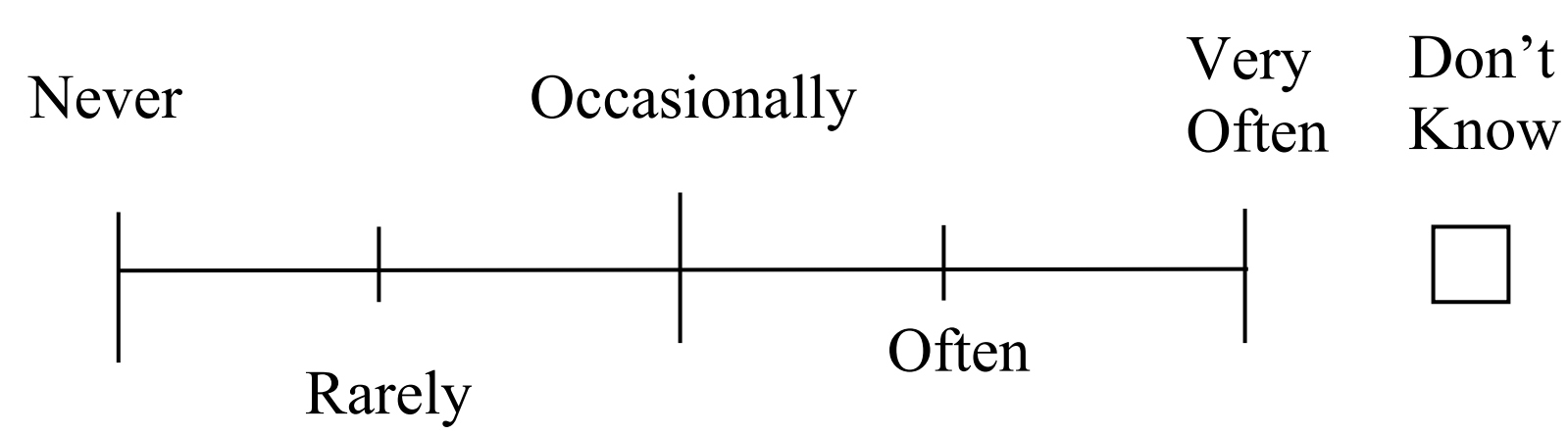

Baseline assessment:
***If item is rated anything higher than "never" ask:**
1. To your knowledge, how has this affected the participant's ability to carry out everyday activities?
2. To your knowledge, does this bother the participant? If yes, how so?

Immediate follow-up assessment:
***If any reported change (improvement or deterioration) from baseline rating, say:**
1. So, before the participant used PTM, your rating was "xx". Now you've said it is "xx". Can you tell me more about this change in rating?

One month follow-up assessment:
***If any reported change (improvement or deterioration) from immediate follow-up rating, ask:**
1. So, when the participant just finished using PTM, your rating was "xx". Now you've said it is "xx". Can you tell me more about this change in rating?

© 2016 Gail Eskes. Not to be used without permission. Electronic copies may be obtained from gail.eskes@dal.ca
Modified by Anne Sophie Champod and Catrina MacPhee with permission.

	Never	Rarely	Occasionally	Often	Very Often	Don't Know
2. Misses left turns while walking or using a wheelchair						☐

Baseline assessment:
***If item is rated anything higher than "never" ask:**
1. To your knowledge, how has this affected the participant's ability to carry out everyday activities?
2. To your knowledge, does this bother the participant? If yes, how so?

Immediate follow-up assessment:
***If any reported change (improvement or deterioration) from baseline rating, say:**
1. So, before the participant used PTM, your rating was "xx". Now you've said it is "xx". Can you tell me more about this change in rating?

One month follow-up assessment:
***If any reported change (improvement or deterioration) from immediate follow-up rating, ask:**
1. So, when the participant just finished using PTM, your rating was "xx". Now you've said it is "xx". Can you tell me more about this change in rating?

	Never	Rarely	Occasionally	Often	Very Often	Don't Know
3. Needs reminders or supervision to move the left foot safely while walking or climbing stairs						☐

Baseline assessment:
***If item is rated anything higher than "never" ask:**
1. To your knowledge, how has this affected the participant's ability to carry out everyday activities?
2. To your knowledge, does this bother the participant? If yes, how so?

Immediate follow-up assessment:
***If any reported change (improvement or deterioration) from baseline rating, say:**
1. So, before the participant used PTM, your rating was "xx". Now you've said it is "xx". Can you tell me more about this change in rating?

One month follow-up assessment:
***If any reported change (improvement or deterioration) from immediate follow-up rating, ask:**
1. So, when the participant just finished using PTM, your rating was "xx". Now you've said it is "xx". Can you tell me more about this change in rating?

© 2016 Gail Eskes. Not to be used without permission. Electronic copies may be obtained from gail.eskes@dal.ca
Modified by Anne Sophie Champod and Catrina MacPhee with permission.

	Never	Rarely	Occasionally	Often	Very Often	Don't Know
4. Forgets to lock left brake, if using a wheelchair						☐

Baseline assessment:
***If item is rated anything higher than "never" ask:**
1. To your knowledge, how has this affected the participant's ability to carry out everyday activities?
2. To your knowledge, does this bother the participant? If yes, how so?

Immediate follow-up assessment:
***If any reported change (improvement or deterioration) from baseline rating, say:**
1. So, before the participant used PTM, your rating was "xx". Now you've said it is "xx". Can you tell me more about this change in rating?

One month follow-up assessment:
***If any reported change (improvement or deterioration) from immediate follow-up rating, ask:**
1. So, when the participant just finished using PTM, your rating was "xx". Now you've said it is "xx". Can you tell me more about this change in rating?

Scanning in Space

	Never	Rarely	Occasionally	Often	Very Often	Don't Know
5. Misses objects on the left side (e.g., on a shelf, or in the fridge)						☐

Baseline assessment:
***If item is rated anything higher than "never" ask:**
1. To your knowledge, how has this affected the participant's ability to carry out everyday activities?
2. To your knowledge, does this bother the participant? If yes, how so?

Immediate follow-up assessment:
***If any reported change (improvement or deterioration) from baseline rating, say:**
1. So, before the participant used PTM, your rating was "xx". Now you've said it is "xx". Can you tell me more about this change in rating?

One month follow-up assessment:
***If any reported change (improvement or deterioration) from immediate follow-up rating, ask:**
1. So, when the participant just finished using PTM, your rating was "xx". Now you've said it is "xx". Can you tell me more about this change in rating?

© 2016 Gail Eskes. Not to be used without permission. Electronic copies may be obtained from gail.eskes@dal.ca
Modified by Anne Sophie Champod and Catrina MacPhee with permission.

6. Misses items on the left side of the plate

Never — Rarely — Occasionally — Often — Very Often | Don't Know ☐

Baseline assessment:
***If item is rated anything higher than "never" ask:**
1. To your knowledge, how has this affected the participant's ability to carry out everyday activities?
2. To your knowledge, does this bother the participant? If yes, how so?

Immediate follow-up assessment:
***If any reported change (improvement or deterioration) from baseline rating, say:**
1. So, before the participant used PTM, your rating was "xx". Now you've said it is "xx". Can you tell me more about this change in rating?

One month follow-up assessment:
***If any reported change (improvement or deterioration) from immediate follow-up rating, ask:**
1. So, when the participant just finished using PTM, your rating was "xx". Now you've said it is "xx". Can you tell me more about this change in rating?

7. Misses the left side of sentences or signs when reading

Never — Rarely — Occasionally — Often — Very Often | Don't Know ☐

Baseline assessment:
***If item is rated anything higher than "never" ask:**
1. To your knowledge, how has this affected the participant's ability to carry out everyday activities?
2. To your knowledge, does this bother the participant? If yes, how so?

Immediate follow-up assessment:
***If any reported change (improvement or deterioration) from baseline rating, say:**
1. So, before the participant used PTM, your rating was "xx". Now you've said it is "xx". Can you tell me more about this change in rating?

One month follow-up assessment:
***If any reported change (improvement or deterioration) from immediate follow-up rating, ask:**
1. So, when the participant just finished using PTM, your rating was "xx". Now you've said it is "xx". Can you tell me more about this change in rating?

© 2016 Gail Eskes. Not to be used without permission. Electronic copies may be obtained from gail.eskes@dal.ca
Modified by Anne Sophie Champod and Catrina MacPhee with permission.

Self-Care Issues

Never | Rarely | Occasionally | Often | Very Often | Don't Know

8. Needs reminders to wash or shave on the left side of the body ☐

Baseline assessment:
***If item is rated anything higher than "never" ask:**
1. To your knowledge, how has this affected the participant's ability to carry out everyday activities?
2. To your knowledge, does this bother the participant? If yes, how so?

Immediate follow-up assessment:
***If any reported change (improvement or deterioration) from baseline rating, say:**
1. So, before the participant used PTM, your rating was "xx". Now you've said it is "xx". Can you tell me more about this change in rating?

One month follow-up assessment:
***If any reported change (improvement or deterioration) from immediate follow-up rating, ask:**
1. So, when the participant just finished using PTM, your rating was "xx". Now you've said it is "xx". Can you tell me more about this change in rating?

Never | Rarely | Occasionally | Often | Very Often | Don't Know

9. Has trouble dressing the left side of the body (e.g., finding it difficult to pull up pants on the left side, or to put the left arm in the sleeve) ☐

Baseline assessment:
***If item is rated anything higher than "never" ask:**
1. To your knowledge, how has this affected the participant's ability to carry out everyday activities?
2. To your knowledge, does this bother the participant? If yes, how so?

Immediate follow-up assessment:
***If any reported change (improvement or deterioration) from baseline rating, say:**
1. So, before the participant used PTM, your rating was "xx". Now you've said it is "xx". Can you tell me more about this change in rating?

One month follow-up assessment:
***If any reported change (improvement or deterioration) from immediate follow-up rating, ask:**
1. So, when the participant just finished using PTM, your rating was "xx". Now you've said it is "xx". Can you tell me more about this change in rating?

© 2016 Gail Eskes. Not to be used without permission. Electronic copies may be obtained from gail.eskes@dal.ca
Modified by Anne Sophie Champod and Catrina MacPhee with permission.

10. Needs reminders to keep the left arm in the correct place

Never	Rarely	Occasionally	Often	Very Often	Don't Know
					☐

Baseline assessment:
***If item is rated anything higher than "never" ask:**
1. To your knowledge, how has this affected the participant's ability to carry out everyday activities?
2. To your knowledge, does this bother the participant? If yes, how so?

Immediate follow-up assessment:
***If any reported change (improvement or deterioration) from baseline rating, say:**
1. So, before the participant used PTM, your rating was "xx". Now you've said it is "xx". Can you tell me more about this change in rating?

One month follow-up assessment:
***If any reported change (improvement or deterioration) from immediate follow-up rating, ask:**
1. So, when the participant just finished using PTM, your rating was "xx". Now you've said it is "xx". Can you tell me more about this change in rating?

11. How much of an effect do left-sided symptoms have on this participant's daily functioning?

1	2	3	4	5	6	7	8	9	10
no disabling effect									a severely disabling effect

We've been focusing on some specific areas that may have been affected by left-sided symptoms, but now we want to hear from you about anything else you may have noticed.

BASELINE ASSESSMENT QUESTIONS:

1. What other changes have you noticed since the participant had the stroke?
2. To your knowledge, how have these changes impacted the participant's everyday life?
3. To your knowledge, do these changes bother the participant? If yes, how so?
4. How have these changes impacted your experience as a caregiver?

© 2016 Gail Eskes. Not to be used without permission. Electronic copies may be obtained from gail.eskes@dal.ca
Modified by Anne Sophie Champod and Catrina MacPhee with permission.

IMMEDIATE POST-TRAINING ASSESSMENT QUESTIONS:

1. To your knowledge, since the participant started using the Peg-the-Mole treatment, what changes have you noticed in their life-sided symptoms and in their ability to carry out everyday activities?
2. To your knowledge, since the participant started using the Peg-the-Mole treatment, what other changes in their daily life have you noticed?
3. How have these changes impacted your experience as a caregiver?

1-MONTH FOLLOW-UP ASSESSMENT QUESTIONS:

1. To your knowledge, since the participant finished using the Peg-the-Mole treatment, what changes have you noticed in their left-sided symptoms and in their ability to carry out everyday activities?
2. To your knowledge, since the participant finished using the Peg-the-Mole treatment, what other changes in their daily life have you noticed?
3. How have these changes impacted your experience as a caregiver

© 2016 Gail Eskes. Not to be used without permission. Electronic copies may be obtained from gail.eskes@dal.ca
Modified by Anne Sophie Champod and Catrina MacPhee with permission.

APPENDIX O
Participant Exit Interview

PARTICIPANT EXIT INTERVIEW

Thank you for taking part in our research study. We are trying to improve treatment for people like you who have had a stroke and would really appreciate your feedback on the therapy you had. Your feedback will be used in our review of the therapy procedures.

Please indicate your level of agreement with each of the following statements:

REGARDING THE THERAPY SESSIONS

I enjoyed using Peg-the-Mole very much.

Not at all true 1	2	3	Somewhat true 4	5	6	Very true 7
**Comments:**						

Peg-the-Mole was fun to use.

Not at all true 1	2	3	Somewhat true 4	5	6	Very true 7
**Comments:**						

I thought Peg-the-Mole was a boring activity.

Not at all true 1	2	3	Somewhat true 4	5	6	Very true 7
**Comments:**						

Peg-the-Mole did not hold my attention at all.

Not at all true 1	2	3	Somewhat true 4	5	6	Very true 7
**Comments:**						

I would describe Peg-the-Mole as very interesting.

Not at all true 1	2	3	Somewhat true 4	5	6	Very true 7
**Comments:**						

I thought Peg-the-Mole was quite enjoyable.

Not at all true 1	2	3	Somewhat true 4	5	6	Very true 7
**Comments:**						

While I was using Peg-the-Mole, I was thinking about how much I enjoyed it.

Not at all true 1	2	3	Somewhat true 4	5	6	Very true 7
**Comments:**						

I think I am pretty good at Peg-the-Mole.

Not at all true			Somewhat true			Very true

1	2	3	4	5	6	7
**Comments:**						

I think I did pretty well at using Peg-the-Mole compared to other participants.

Not at all true<br>1	2	3	Somewhat true<br>4	5	6	Very true<br>7
**Comments:**						

After working at using Peg-the-Mole for awhile, I felt pretty competent.

Not at all true<br>1	2	3	Somewhat true<br>4	5	6	Very true<br>7
**Comments:**						

I am satisfied with my performance at using Peg-the-Mole.

Not at all true<br>1	2	3	Somewhat true<br>4	5	6	Very true<br>7
**Comments:**						

I was pretty skilled at using Peg-the-Mole.

Not at all true<br>1	2	3	Somewhat true<br>4	5	6	Very true<br>7
**Comments:**						

Peg-the-Mole was an activity that I could not do very well.

Not at all true 1	2	3	Somewhat true 4	5	6	Very true 7
**Comments:**						

The instructions during Peg-the-Mole were easy to follow.

Not at all true 1	2	3	Somewhat true 4	5	6	Very true 7
**Comments:**						

The pictures and instructions on the iPad screen during Peg-the-Mole were hard to see or read.

Not at all true 1	2	3	Somewhat true 4	5	6	Very true 7
**Comments:**						

I put a lot of effort into using Peg-the-Mole.

Not at all true 1	2	3	Somewhat true 4	5	6	Very true 7
**Comments:**						

I did not try very hard to do well at Peg-the-Mole.

Not at all true 1	2	3	Somewhat true 4	5	6	Very true 7

**Comments:**						

I tried very hard on Peg-the-Mole.

Not at all true 1	2	3	Somewhat true 4	5	6	Very true 7
**Comments:**						

It was important to me to do well at Peg-the-Mole.

Not at all true 1	2	3	Somewhat true 4	5	6	Very true 7
**Comments:**						

I did not put much energy into using Peg-the-Mole.

Not at all true 1	2	3	Somewhat true 4	5	6	Very true 7
**Comments:**						

I did not feel nervous at all while using Peg-the-Mole.

Not at all true 1	2	3	Somewhat true 4	5	6	Very true 7
**Comments:**						

I felt very tense while using Peg-the-Mole.

Not at all true 1	2	3	Somewhat true 4	5	6	Very true 7
**Comments:**						

I was very relaxed while using Peg-the-Mole.

Not at all true 1	2	3	Somewhat true 4	5	6	Very true 7
**Comments:**						

I was anxious while using Peg-the-Mole.

Not at all true 1	2	3	Somewhat true 4	5	6	Very true 7
**Comments:**						

I felt pressured while using Peg-the-Mole.

Not at all true 1	2	3	Somewhat true 4	5	6	Very true 7
**Comments:**						

Pointing to the iPad screen during Peg-the-Mole was comfortable.

Not at all true 1	2	3	Somewhat true 4	5	6	Very true 7
**Comments:**						

The speed of the targets during Peg-the-Mole was about right.

Not at all true 1	2	3	Somewhat true 4	5	6	Very true 7
**Comments:**						

I believe I had some choice about using Peg-the-Mole.

Not at all true 1	2	3	Somewhat true 4	5	6	Very true 7
**Comments:**						

I felt like it was not my own choice to use Peg-the-Mole.

Not at all true 1	2	3	Somewhat true 4	5	6	Very true 7
**Comments:**						

I did not really have a choice about using Peg-the-Mole.

Not at all true 1	2	3	Somewhat true 4	5	6	Very true 7
**Comments:**						

I felt like I had to use Peg-the-Mole.

Not at all true 1	2	3	Somewhat true 4	5	6	Very true 7

**Comments:**						

I used Peg-the-Mole because I had no choice.

Not at all true 1	2	3	Somewhat true 4	5	6	Very true 7
**Comments:**						

I used Peg-the-Mole because I wanted to.

Not at all true 1	2	3	Somewhat true 4	5	6	Very true 7
**Comments:**						

I used Peg-the-Mole because I had to.

Not at all true 1	2	3	Somewhat true 4	5	6	Very true 7
**Comments:**						

I believe Peg-the-Mole could be of some value to me.

Not at all true 1	2	3	Somewhat true 4	5	6	Very true 7
**Comments:**						

I would be willing to use Peg-the-Mole again because it has some value to me.

Not at all true 1	2	3	Somewhat true 4	5	6	Very true 7
**Comments:**						

I believe using Peg-the-Mole could be beneficial to me.

Not at all true 1	2	3	Somewhat true 4	5	6	Very true 7
**Comments:**						

I think Peg-the-Mole is an important activity.

Not at all true 1	2	3	Somewhat true 4	5	6	Very true 7
**Comments:**						

GENERAL QUESTIONS ABOUT THE STUDY

What did you like *the most* about Peg-the-Mole?
What did you like *the least* about Peg-the-Mole?

What did you find the most challenging when using Peg-the-Mole?
Is there anything that has been particularly helpful about participating in this study?
Is there anything that has been disappointing about participating in this study?
What change(s) could make Peg-the-Mole more enjoyable to use?
Would you be willing to continue using Peg-the-Mole at home?
Would you recommend Peg-the-Mole to other people who have had a stroke like yours?

APPENDIX P
Training log

Participant ID: ______________________

Participant Training Log

Session	Date	PTM Start Time / Stop Time	Comments:	Completed? (✓)	
				Pre-Game	Post-Game
1					
2					
3					
4					
5					
6					
7					
8					
9					
10					

APPENDIX S
Assessment table

Screening Measures (Given before baseline assessment)

Function being measured	Test name	Test Description	Time to complete
General physical and cognitive health	Health History self-report questionnaire	Participants will be asked over the phone about their general health such as major illnesses or medical events.	10 min
Multidimensional stroke outcomes	Stroke Impact Scale – Short Form	A brief, 8-item self-report questionnaire that assesses stroke impact in 8 domains: strength, hand function, ADLs, mobility, communication, emotion, memory and thinking, and participation. Lower scores indicate greater impact.	2 min
**Total approximate time for screening measures:**			**12 min**

Baseline Measures (Given before treatment begins to determine clinical trial eligibility)

Function being measured	Test name	Test Description	Time to complete
General cognitive health	Judgement of Line Orientation (Benton et al., 1983), modified by Woodard et al., 1998).	A measure of visuospatial judgement and awareness. Participants are asked to indicate which two lines on the bottom of the page are in the exact same position and point in the same direction as two lines on the top of the page. (Woodard et al., 1998).	5 min
	Visual Confrontation Test	A brief visual field exam. Participants are asked to follow a clinician's hand (with one eye blocked) and indicate how many fingers are being held up. The sequence is repeated for both eyes and all four quadrants of the visual field.	2 min
	The Montreal Cognitive Assessment (MoCA; Nasreddine et al., 2005)	Screening test of overall cognitive abilities. Participants are asked to complete tasks such as drawing a clock, naming animals, and repeating sentences.	5 min
	Digit Span (forward and backward)	A test of short-term verbal memory. Participants are asked to repeat strings of verbally presented numbers forward and backward.	5 min

	Trail Making Test (Reitan, 1958)	A test of psychomotor speed (ability to search and shift between automatic sequences). Participants are asked to rapidly draw lines connecting numbers and letters in order.	5 min
Tests assessing neglect	Attention Motor Task (Husain et al., 2000)	A reaching task performed on a touchscreen computer. Participants are simply asked to reach and to touch one of the indicated target buttons, while starting from different positions.	20 min
	Abbreviated Landmark Task (Bisiach et al., 1998)	A task used to classify neglect into a primarily attentional/perceptual deficit or premotor deficit. The participant makes perceptual judgements about pre-transected horizontal lines.	5 min<br>**47 min**

Post PTM Session (given immediately after each prism session)

**Function being measured**	**Test name**	**Test Description**	**Time to complete**
After-effects of prism adaptation	Modified Proprioceptive-and-Visual Pointing Outcome Task	A modified proprioceptive-and-visual straight-ahead outcome task. Ten trials will be administered immediately before and after each training session. Participants will be asked to reach and touch one of two locations that a mole appeared. (i.e., perform memory-guided pointing movements).	10 min
**Total approximate time for testing session (including set-up, outcome task, intervention, and training logs):**			**30 min**

Effectiveness Outcome Measures (given before treatment begins, after the last PTM session, and one-month post-treatment)

**Function being measured**	**Test name**	**Test Description**	**Time to complete**
Tests assessing neglect	Behavioural Inattention Test (BIT) – (Wilson et al., 1987)	Includes subtests that measure neglect severity, including line crossing, letter cancellation, star cancellation, figure and shape copying, line bisection, representational drawing, menu reading, article reading, and picture scanning.	20 min

	Eschenbeck Standardized Activity of Daily Living (SADL) Test: Filling out a Form (Eschenbeck et al., 2010)	Participants are asked to fill out a form that contains fields boxes in the left, right, and center of the page.	5 min
	Halifax Neglect Impact Scale (Patient and Caregiver versions)	Includes questions assessing the frequency of neglect symptoms seen in activities of daily living.	10 min
Tests assessing mental health	Hospital Anxiety and Depression Scale (Zigmond & Snaith, 1983)	A self-report measure of anxiety and depression symptoms.	2.5 min
	Fatigue Severity Scale (Krupp et al., 1989)	A self-report measure of the severity of fatigue and its effect on daily activities.	2.5 min
**Total approximate time for outcome assessment measures:**			**40 min**

Exit Questionnaire (given after the last PTM session)

Function being measured	Test name	Test Description	Time to complete
Participant Feedback	Modified Intrinsic Motivation Inventory (Ryan & Deci, 2000)	A questionnaire for assessing the subjective experience of participants regarding the usability of our novel iPad application. Additional questions were added.	20 min
**Total approximate time for exit questionnaire:**			**20 min**

APPENDIX T
Home Visit Safety Checklist

Page 1

Home Visit Safety Checklist

Please complete the survey below.

Thank you!

1)	Are there pets in the home?	◯ Yes<br>◯ No<br>(If yes, please place pet in another room upon study staff arrival.)
2)	Are there firearms in the home?	◯ Yes<br>◯ No<br>(If yes, please ensure they are locked up and free of ammunition.)
3)	Do you or anyone else smoke in the home?	◯ Yes<br>◯ No<br>(If yes, please refrain from smoking while study staff are present in the home.)
4)	Do you currently have problems with bedbugs or other types of infestation?	◯ Yes<br>◯ No
5)	Who do you expect to be present for the visit?	__________________________________
6)	Are there any other safety concerns we should be aware of?	__________________________________
7)	Do you have a table or surface area that can fit two people and some study equipment (e.g., paper, iPad, etc.)?	◯ Yes<br>◯ No

APPENDIX U

Baseline Results Summary

Engaging Mobile Prism Adaptation Treatment at Home (EMPATH)

Neuropsychological Results Summary

Investigators: Dr. Anne Sophie Champod (Principle Investigator), Dr. Gail Eskes (Co-Investigator), Catrina MacPhee

Participant code: ___________________________ Date of testing: _________________________

Test Administrator: ________________________ Place of testing: ________________________

**Neuropsychological Test**	**What is measured**	**Performance**
The Montreal Cognitive Assessment	Screening test of overall cognitive abilities	
Judgement of Line Orientation	Measure of visuospatial judgement and awareness	
Digit span (forward and backward)	Measure of short-term verbal memory<br>(ability to keep numbers in mind for a short while)	
Trail Making Test	Measure of psychomotor speed<br>(ability to search and shift between automatic sequences)	
Behavioural Inattention Test	Subtests to assess the impact of visual neglect on functional tasks	
Fatigue Severity Scale	Self-report questionnaire that measures the severity of fatigue and its effect on activities	
Hospital Anxiety and Depression Scale	Self-report measure used to detect states of depression and anxiety	
Comments:		

These tests were conducted for the purpose of the above research study and are not intended to take the place of a clinical neuropsychological assessment. If more information is required, please contact
Dr. Anne Sophie Champod: 902-585-1332 or anne.champod@acadiau.ca
Dr. Gail Eskes: 902-494-4033 or gail.eskes@dal.ca

APPENDIX V

Final Results Summary

Engaging Mobile Prism Adaptation Treatment at Home (EMPATH)

Neuropsychological Results Summary

Investigators: Dr. Anne Sophie Champod (Principle Investigator), Dr. Gail Eskes (Co-Investigator), Catrina MacPhee

Participant code: ___________________________ Date of testing: _________________________

Test Administrator: ________________________ Place of testing: ________________________

**Neuropsychological Test**	**What is measured**	**Performance**
Behavioural Inattention Test	Subtests to assess the impact of visual neglect on functional tasks	
Fatigue Severity Scale	Self-report questionnaire that measures the severity of fatigue and its effect on activities	
Hospital Anxiety and Depression Scale	Self-report measure used to detect states of depression and anxiety	
Comments:		

These tests were conducted for the purpose of the above research study and are not intended to take the place of a clinical neuropsychological assessment. If more information is required, please contact
Dr. Anne Sophie Champod: 902-585-1332 or anne.champod@acadiau.ca
Dr. Gail Eskes: 902-494-4033 or gail.eskes@dal.ca

Appendix W

Daily Game Instructions and Checklist

Set-up Checklist

- ☐ Place the equipment where the tape is on the table.
- ☐ Make sure the iPad is placed on the Velcro pieces connected to the mat.
- ☐ Click on the Peg-the-Mole app to launch it.
- ☐ Sit upright in front of the iPad.
- ☐ Make sure your body is centered with the keyboard and the iPad screen.
- ☐ Extend your arm to touch the screen and adjust chair so there is a slight bend in your arm.
- ☐ Use string attached to the back of the iPad to measure the distance you should be sitting away from the iPad. The string should reach your shoulder when pulled tight.
- ☐ Follow the instructions for the pre-game **(no goggles)**
- ☐ Follow the instructions for the PTM practice **(no goggles)**
- ☐ Follow the instructions for the PTM game **(put goggles on!)**
- ☐ Follow the instructions for the post-game **(remove goggles!)**
- ☐ Complete the training log for today (online through email link or paper copy)

Instructions Reminder:

<u>Pre-Game Instructions:</u>

NO GOGGLES.

1. I set up my equipment before I begin the pre-game *(refer to the set-up checklist)*.
2. After I check that my equipment is set up correctly, I press “start” on the screen to begin today’s session.
3. After I read or listen to the instructions for the pre-game, I press “s” on my keyboard to start.
4. When the pre-game starts, I wait for a target to appear. Once the target appears, I remember where the target is located. Once I am ready for the target to disappear, I hold the space bar down to make the target disappear behind a colour cloud.
5. I leave my index finger on the space bar until the colour cloud goes away.

6. After the colour cloud goes away, I touch the screen where I think the target was before it disappeared.
7. After I touch the screen and hear a "ding", I return my finger to the space bar.
8. I wait for the next target to appear before I press the space bar down. Once I am ready for the target to disappear, I hold the space bar down.
9. I repeat all steps until the screen shows: "Press C to continue"

Peg-the-Mole Practice Instructions

NO GOGGLES.

1. I press the "P" key on the keyboard to begin the game practice.
2. After I'm done practicing, I press "C" to continue.

Peg-the-Mole Game Instructions

GOGGLES.

1. I close my eyes and put the goggles on.
2. I press the "S" key on the keyboard to start the game.
3. During this game I hold the space bar down. I will then see a mole appear on the screen holding a target. As soon as the mole squeaks, I let go of the space bar and hit the bull's eye on the screen as quickly as I can. Make sure to touch the screen even if the mole disappeared before you were able to do so.
4. I am only using ONE HAND to play the game.
5. If the game freezes, try touching the screen and then coming back to the space bar to start the next trial.
6. Once the game is over, I listen to the instructions for the Post-Game with my **goggles on.**

Post-game Instructions:

NO GOGGLES

1. Once I have listened to the instructions for the Post-Game, I close my eyes and **remove my goggles.**

2. I wait for a target to appear. Once the target appears, I remember where the target is located. Once I am ready for the target to disappear, I hold the space bar down to make the target disappear behind a colour cloud.
3. I leave my index finger on the space bar until the colour cloud goes away.
4. After the colour cloud goes away, I touch the screen where I think the target was before it disappeared.
5. After I touch the screen and hear a "ding", I return my finger to the space bar.
6. I wait for the next target to appear before I press the space bar down. Once I am ready for the target to disappear, I hold the space bar down.
7. I repeat all steps until the screen shows the scoreboard.
8. Once the game has finished, I press "~" on the keyboard to exit.
9. I complete my daily training log (online via an email link or paper copy).

APPENDIX X

Connecting Bluetooth Keyboard and iPad Instructions

Turn Bluetooth keyboard "on" (on/off switch is located on the back of the keyboard). Once the keyboard is turned on, it should automatically pair with the iPad. If the keyboard does not automatically pair with the iPad, please try the following steps:

1. Launch the iPad's settings by finding the grey wheel icon labelled "settings" on the iPad's home screen. Then, click "Bluetooth".

2. Once you press the "Bluetooth" settings, ensure the "Bluetooth" setting is toggled to "on".

3. On the back of the keyboard, press and hold the "connect" push button to make the keyboard discoverable to the iPad. As you are holding down the "connect button" you should see a flashing green light on the front of the keyboard.

4. The keyboard should then appear as an available Bluetooth device on your iPad screen. The Bluetooth keyboard may show up under "my devices" or under "other devices". Once you see the keyboard show up as an available Bluetooth device, press the "Bluetooth Keyboard" button that appears on the iPad.

5. A code will appear on the iPad screen. Please type the code number you see on the screen using the keyboard. You **will not** see the numbers appear on the screen as you type them – that is normal.

6. Once the code has been entered, press "enter" on the keyboard. The keyboard should now be successfully paired to the iPad. Repeat steps if the pairing was unsuccessful.

APPENDIX Y

Peg-the-Mole Gameplay Observation Checklist

SET-UP			
	Yes	*No*	*Comments:*
Places iPad and keyboard back within the tape marks?			
Keyboard is turned on?			
If keyboard is turned off, is able to turn it on?			
Has slight bent arm when touches the screen?			
Launches the Peg-the-Mole app independently?			
**PRE-GAME**			
	Yes	*No*	*Comments:*
Wears goggles? (should be no goggles)			
Waits for colour mask to disappear before touching screen?			
Readily returns finger to space bar? (Delay? Forgets?)			
Makes a single touch to the screen?			
Any other relevant notes			
**PTM GAME**			
	Yes	*No*	*Comments:*
Remembers "no goggles" for practice?			
Puts their goggles on before pressing "s" for exposure trials?			
Closes their eyes to put goggles on?			
Engages in any unusual behaviours with the goggles that could impact performance (e.g., looking over the goggles, taking them off and on, odd head movements)?			
Gets distracted by the environment?			
Returns finger to keyboard?			
Attempts to self-correct (e.g., slowing down to improve accuracy)?			
Any other relevant notes			
**POST-GAME**			
	Yes	*No*	*Comments:*
Wears goggles? (should be no goggles)			
Waits for colour mask to disappear before touching screen?			
Readily returns finger to space bar? (Delay? Forgets?)			
Makes a single touch to the screen?			
Any other relevant notes			
